# Enhanced optimization-based method for the generation of patient-specific models of Purkinje networks

**DOI:** 10.1038/s41598-023-38653-1

**Published:** 2023-07-21

**Authors:** Lucas Arantes Berg, Bernardo Martins Rocha, Rafael Sachetto Oliveira, Rafael Sebastian, Blanca Rodriguez, Rafael Alves Bonfim de Queiroz, Elizabeth M. Cherry, Rodrigo Weber dos Santos

**Affiliations:** 1 Graduate Program in Computational Modeling, Federal University of Juiz de Fora, Juiz de Fora, Brazil; 2 Department of Computer Science, Federal University of São João del-Rei, São João del-Rei, Brazil; 3 Department of Computer Science, Federal University of Ouro Preto, Ouro Preto, Brazil; 4 School of Computational Science and Engineering, Georgia Institute of Technology, Atlanta, GA USA; 5 Department of Computer Science, Universitat de Valencia, Valencia, Spain; 6 Department of Computer Science, University of Oxford, Oxford, UK

**Keywords:** Computational biology and bioinformatics, Cardiology

## Abstract

Cardiac Purkinje networks are a fundamental part of the conduction system and are known to initiate a variety of cardiac arrhythmias. However, patient-specific modeling of Purkinje networks remains a challenge due to their high morphological complexity. This work presents a novel method based on optimization principles for the generation of Purkinje networks that combines geometric and activation accuracy in branch size, bifurcation angles, and Purkinje-ventricular-junction activation times. Three biventricular meshes with increasing levels of complexity are used to evaluate the performance of our approach. Purkinje-tissue coupled monodomain simulations are executed to evaluate the generated networks in a realistic scenario using the most recent Purkinje/ventricular human cellular models and physiological values for the Purkinje-ventricular-junction characteristic delay. The results demonstrate that the new method can generate patient-specific Purkinje networks with controlled morphological metrics and specified local activation times at the Purkinje-ventricular junctions.

## Introduction

The ventricular conduction system (VCS) is an essential part of the heart since it is responsible for synchronous stimulation of the ventricular walls and is crucial for the proper maintenance of the heart rhythm. The VCS comprises the His-bundle, left and right bundles branches, and the Purkinje fiber network (PN). The system provides ventricular contraction from apex to base, together with synchronization of the left and right ventricles, which allows the correct coordination of electrical activity in mammalian species^1^.

In the current clinical and modeling literature, several studies have implicated the PN as both trigger and maintainer of deadly cardiac arrhythmias, such as ventricular fibrillation (VF)^2–5^. Furthermore, it has been shown that Purkinje cells and ventricular cells have different electrophysiological properties^6,7^. For instance, Purkinje cells’ action potentials (APs) have a faster depolarization and a more negative plateau phase. This characteristic results in longer action potential duration (APD) when compared to ventricular cells^8^, which makes Purkinje cells more susceptible to developing pro-arrhythmic abnormalities, like early-after-depolarizations and delayed after-depolarization. This contrast in the APD form is likely apparent at the Purkinje-ventricular-junction (PVJ) sites. Due to the distinct shape of the action potential of Purkinje and ventricular cells, electrotonic effects and APD dispersion occur between the cells from the Purkinje fiber and those from the ventricular tissue^6^. In addition, at the PVJ sites, a characteristic delay in conduction, 3–5 ms, is observed from the Purkinje to the ventricular tissue. Mainly, this phenomenon arises when a small volume (i.e., the source, Purkinje cells) tries to depolarize a large volume (i.e., the sink, many connected ventricular cells) and is also referred to as source-sink mismatch^9–11^. Therefore, to properly study cardiac electrophysiology via computational modeling, reliable models of the PN are needed and must take into account both morphological and electrical properties.

Personalized models of cardiac electrophysiology that match clinical observation with high fidelity, referred to as cardiac digital twins (CDTs), are promising tools for tailoring cardiac precision therapies^12^. An essential step towards CDTs relies on the ability of models to replicate the ventricular activation sequence under a broad range of conditions. However, despite recent progress on cardiac functional and imaging techniques, there is not a valid technique to extract a PN from clinical data, which makes the development of a coupled Purkinje-ventricular patient-specific computational model challenging^13,14^. Therefore, as a result of their complex geometry, manual generation of PNs can become a highly demanding and time-consuming task^15^, making the development of methods that can automatically generate PNs a requirement to advance studies in this field.

Purkinje networks are known to have a complex morphology with several branches and bifurcations, as can be seen in physiological images^16–19^. A wide variety of methods have been used to address the generation of realistic PNs. In particular, PNs can be generated using image processing techniques by extracting the structure from images of dissected ventricles and them projecting these flat networks onto realistic endocardial surfaces^17^. Such models gather both the geometry and the local activation time (LAT) from the processed network. On the other hand, the generated PN may not be acceptable for other subjects due to biological variability. Another set of widely utilized methods for PN generation is fractal trees. In such models, the L-System is commonly described as an alternative to automatically produce PNs using a pre-defined set of rules that not only can prevent collisions^20^ but also enhance its geometry^21^. An alternative to the L-System is the fractal method proposed by Costabal et al.^22^ which allows the automatic generation of PNs using controllable curvature of the branches, enhancing the geometry of the tree, especially in irregular surfaces. Moreover, fractal trees and image processing techniques can be combined to construct the VCS. For instance, in the work of Bordas et al.^23^, from magnetic resonance images of a rabbit, it was possible to identify the Purkinje network partially. After manually constructing the His-Bundle, a fractal method was applied to extend the PN to the myocardium. Furthermore, there are models which are LAT constrained. Activation times data is acquired from patients and utilized to grow the networks, like in the work of Vergara et al.^13^ and in the recent study of Barber et al.^24^.

Another class of methods is based on optimization principles^25^ and was inspired by the Constructive Constrained Optimization (CCO) method that can generate detailed and realistic vascular trees^26^. A major advantage of this method is the flexibility of using any cost function in the method’s optimization process and including as many topological and electrical metrics as needed (e.g., bifurcation angles, PVJ location, LAT).

We develop a novel method using optimization principles to generate patient-specific PNs with geometrical and activation accuracy. The new method, named Constructive Optimization (CO), is a modified version of the classical CCO method that is traditionally utilized for the construction of vascular networks. The CO is focused on generating patient-specific PN models and as such it considers a cost functions that relies on electrical and geometrical metrics. Both methods rely on optimization principles to generate a topology. However, their main difference is that the CCO considers vascular concepts in its equations and has a set of constraints that all segments in the tree must fulfill during the optimization process. These restrictions are associated with blood flow and hydrodynamic resistance of the segments from the growing tree, which are not present in the CO method for Purkinje networks.

In this work, we have evaluated the generated PN models by coupling them to biventricular meshes and comparing the LATs obtained in the simulations to different references. The simulations are based on modern cellular electrophysiology models for human Purkinje and ventricular cells and a fast parallel cardiac simulator. Our results show that the generated patient-specific PNs can accurately reproduce important geometrical and electrical features. In addition, the new PN models also correctly reproduced the anterograde physiological delay at the Purkinje-ventricular junctions. Therefore, we believe the results presented in this work are an essential step towards a better understanding of Purkinje networks and provide a valuable tool to study the role of patient-specific models and their impact, for instance, in the simulations of cardiac arrhythmias.

## Methods

### Method overview

Constructive Optimization (CO), a method developed to generate Purkinje networks^25^ in tri-dimensional domains following a cost function that minimizes the total length of the network, is the foundation to develop the PN models of this work. In this work, the new method uses as input a given cloud of points representing the surface to be covered, an initial root position, and a prescribed cost function to be minimized. The method aims to generate a tree with branches that satisfy a set of restrictions and, at the same time, minimize the user-specified cost function.

The novel patient-specific Purkinje generation method (Algorithm 1) can be summarized as follows:

1. To generate a Purkinje network the framework requires an endocardium surface $\Omega _{S}$ containing a set of points *S* with distal locations for the terminal branches and a proximal location of the root branch $x_{prox}$. Optionally, an initial Purkinje network topology can also be provided as starting root of the method.
2. Two different cost functions are used in the optimization procedure. One for placing new intermediate branches, cost function $CF_{i}$, and another for active branches, cost function $CF_{a}$.
3. $CF_{i}$ is used to assure that the PN topology respects the patient-specific anatomy, and other know characteristics of human PN topology, such as segment length, angle between bifurcating segments, among others.
4. $CF_{a}$ is used to assure that the PN reproduces the patient-specific electrophysiology, such as ECG, and endocardial activation maps.
5. A *PreProcessing* subroutine is responsible for dividing the set *S* into two subsets: intermediate points, $S_{i}$, and active PVJs, $S_{a}$. These two subsets are used by the two different cost functions described above.
6. The main loop consists of branch generation using geodesic pathways and the minimization of the two cost functions until a stop criterion is reached.
7. Finally, a *PostProcessing* subroutine connects any remaining active PVJs, via pruning and other techniques.

To emphasize, set *S* contains the locations of the active PVJs, $S_a$, and an extra cloud of intermediate points, $S_i$, that homogeneously cover $\Omega _s$.

In addition, the PN is represented by a graph data structure, where the nodes represent points over the surface, and the edges are segments that link two points. Based on this data structure, each segment has access to its parent and offspring. A terminal segment is defined as a segment with no offspring, and a branch is a set of segments between bifurcations. For more details related to the geometrical concepts and data structures used by the method, please refer to Supplementary Material section A.1.

In the following subsections each step of the method is explained in more detail.

#### Pre-processing steps

Before growing the PN, a *PreProcessing* step is executed on the provided endocardium surface $\Omega _s$. We extract all the points, store them in a set *S*, and divide them into two sets: intermediate and active PVJ points. An intermediate point is mainly used to increase the endocardium coverage of the growing PN with additional branches. On the other hand, an active PVJ point has a corresponding LAT, which is used as a target in the optimization process.

**Figure 1:**
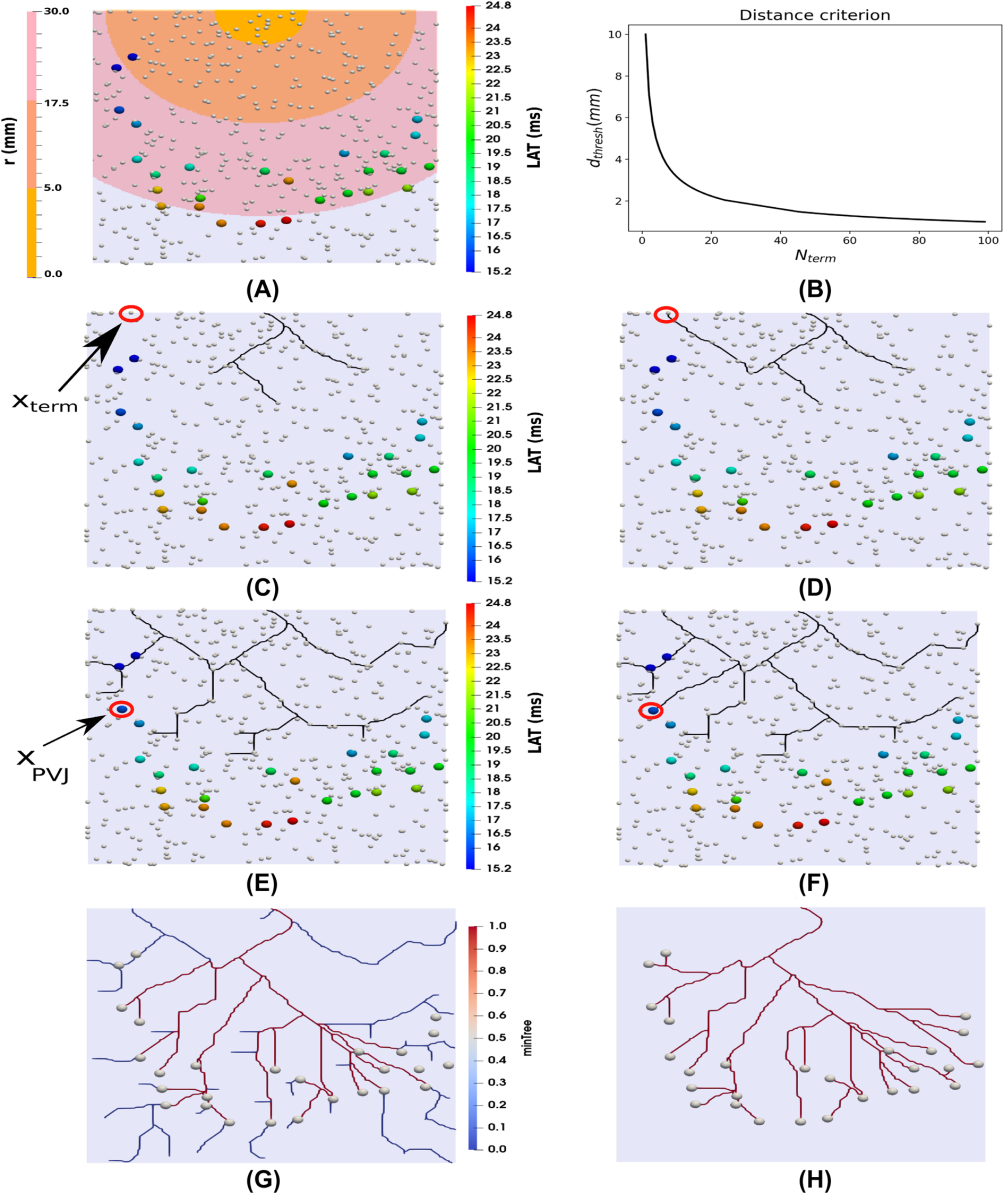
Summary of the method. In panels (**A**) and (**B**), the *PreProcessing* step is highlighted showing how the intermediate points are renumbered and the effect of the distance criterion in the $d_{thresh}$ evolution, as more terminals are added to the PN, respectively. Next in panels (**C**) and (**D**), an example connection for a intermediate terminal, $x_{term}$, is depicted, while in panels (**E**) and (**F**), a connection considering an active PVJ terminal, $x_{PVJ}$, is illustrated. Finally in panels (**G**) and (**H**), the *PostProcessing* step is shown illustrating the pruning of extra branches and connection of the remaining active PVJs.

First, the *PreProcessing* subroutine reorders the indices of the intermediate points in *S*, as illustrated in Fig. 1A. This operation considers the initial root position, $x_{prox}$, passed as an input parameter to reorder the points in *S* so that the numbering of points increases with the distance to the initial root position. The user can also provide an input set of active PVJ points inside the set *S*. In this case, during the *PreProcessing* subroutine, we pre-process the PVJs by sorting them according to their LATs and return these points as set $S_a$.

#### Root placement

After this initial step, the method constructs the root branch, which can be done in two different forms: using only the given initial root position $x_{prox}$ or by providing an initial PN topology.

In the case an initial PN is provided, this structure is used as the initial topology of the tree. This input offers flexibility and advantages, in particular in two scenarios. Firstly, well-known structures of the VCS, like the Left-Bundle-Branch (LBB) and Right-Bundle-Branch (RBB), can be constructed using different techniques and supplied as the initial network. The second advantage of this feature is extending an already created PN by adding other branches to its topology.

Therefore, if the initial PN is not provided, given the initial root position $x_{prox}$, a distal location $x_{term}$ is selected from the intermediate set $S_{i}$ and must satisfy a distance criterion, which is explained next. After selecting a feasible position (one for which the distance criterion is satisfied), a geodesic pathway that connects $x_{prox}$ to $x_{term}$ is constructed over $\Omega _s$.

The distance criterion consists of checking whether the minimum distance $d_{crit}$ of a new candidate terminal location $x_{term}$ to all existing segments exceeds an adaptive threshold ($d_{thresh}$). This threshold is dynamically decreased when the number of terminal branches increases during the process of PN generation. The same 3D formulation utilized in Ulysses et al.^25^ was applied in the present work. Before adding a new terminal branch, the threshold distance $d_{thresh}$ is initially given by:

1 $$\begin{aligned} d_{thresh} = \sqrt{\dfrac{l_{d}^{2}}{k_{term}}}, \end{aligned}$$

where $k_{term}$ is the current number of terminals in the PN and $l_{d}$ is the characteristic length of the domain in millimeters. Figure 1B illustrates how the threshold distance $d_{thresh}$ varies as the number of terminals in the PN, $k_{term}$, increases for $l_{d}=10mm$. It is important to note that as the network grows, the threshold distance decreases as more terminals are connected to the tree. This behaviour leads to longer branches in the early iterations of the method. Consequently, shorter branches start to appear during later iterations.

#### Connection of an intermediate branch

After the root is placed, the method enters the main loop. At each iteration, a point $x_{term}$ from $S_{i}$ is selected, and a new intermediate branch *B* is generated by calling the *GenerateTerminal* subroutine. When selecting the new branch *B*, we try to minimize the total length of the PN. To do so, we select the shortest branch that connects $x_{term}$ to one of the existing segments of the tree, *s*, subject to the following restrictions, $R_i$: the new branch must satisfy the distance criterion, its segments must belong to the endocardial surface and there should be no collisions with other segments in the tree. Therefore, given a point $x_{term}$ from $S_i$ we connect it to a segment *s* of the PN by solving minimization problem (2) alongside the cost function given by Eq. (3):

2 $$\begin{aligned} \min _{s \in PN} CF_i( B (x_{term}, s) ) \\ \text {s.t.} \, B \in R_i, \nonumber \end{aligned}$$

with the cost function

3 $$\begin{aligned} CF_{i} (B) = \sum _{k=0}^{N_{seg}} l_k, \end{aligned}$$

where $l_k$ is the length of each segment *k* of the branch *B*, $N_{seg}$ is the total number of segments in *B*, and the subscript *i* refers to intermediate points. To speed up this minimization process, we use the Euclidean distance as a proxy for the geodesic pathway over $\Omega _s$, as explained next. First, we sort all the segments of the tree by their Euclidean distance to $x_{term}$ and store the closest $N_{i}$ segments. Then, a geodesic pathway algorithm, which is computationally expensive, generates new branches that connect $x_{term}$ to the nearest $N_{i}$ segments. After this step, we choose the shortest branch that attends the distance criterion without collisions. The procedure of connecting an intermediate point is illustrated in Fig.  1C and D.

#### Connection of an active PVJ branch

In the main loop, we attempt to connect the active PVJs present in $S_{a}$ when $k_{term}$ is a multiple of a connection rate parameter, $L_{rate}$, by calling the *AttemptPVJConnection* subroutine. In this scenario, we try to sequentially connect all the unconnected PVJs. For each active PVJ, $x_{PVJ}$, we now look for a new branch $B_a$ that minimizes the difference between the target LAT and the simulated LAT at $x_{PVJ}$, subject to the following restrictions $R_a$: the new branch must satisfy the distance criterion, its segments must belong to the endocardial surface, there should be no collisions with other segments in the tree, and the absolute LAT error must not exceed a given tolerance, $L_{error}$.

To simulate the LATs we use the cable equation, which is commonly used to describe the electrical flow in neurons and cardiac cells^27^. Here, the Purkinje fiber is considered to have a cylindrical shape, with a certain length, diameter, internal conductivity, and membrane capacitance. The conduction velocity (*CV*) across the cable is given by

4 $$\begin{aligned} CV = \sqrt{ \dfrac{G d}{4 C_f \tau _f} }, \end{aligned}$$

where *G* is the internal conductivity, $C_f$ is the membrane capacitance, $\tau _f$ is a time constant, and *d* is the diameter of the cable. To evaluate a new branch in terms of LAT, we temporarily connect that branch to the PN via a segment, *s*. Next, we sum to the current LAT of the segment *s* the times of conduction velocity obtained via the cable equation, of every segment in the pathway between $x_{PVJ}$ and the segment *s*. To speed up the minimization process for selecting branch $B_a$, we use the Euclidean distance as a proxy for the geodesic pathway over $\Omega _s$. First, we estimate the LAT errors of each segment *s* of the PN using the cable equation applied to a straight line that connects *s* and $x_{PVJ}$. Next, we use a geodesic pathway algorithm to compute the $N_a$ branches with the lowest estimates for LAT errors. We temporarily connect each branch to the PN and compute the LAT error using the cable equation. After this step, we choose the branch with the lowest LAT error that satisfies restrictions $R_a$. Therefore, given a active PVJ, $x_{PVJ}$, from $S_a$ we connect it to a segment *s* of the PN by solving minimization problem (5) alongside the cost function given by Eq. (6):

5 $$\begin{aligned} \min _{s \in PN} CF_a( B (x_{PVJ}, s) ) \\ \text {s.t.} \, B \in R_a, \nonumber \end{aligned}$$

with the cost function

6 $$\begin{aligned} CF_{a} = |LAT(s) - T(PVJ)|, \end{aligned}$$

where *s* is the new terminal segment linked to an active PVJ, *LAT*(*s*) is a function that returns the LAT of a given segment *s* using the cable equation, *T*(*PVJ*) is the known value for the LAT of the PVJ, and the subscript *a* refers to active PVJ points. Finally, if at least one PVJ is connected to the PN, the procedure is repeated until there are no updates to the tree. The whole procedure of connecting an active PVJ, $x_{PVJ}$, is illustrated in Fig. 1E and F.

#### Post-processing steps

After we reach the stopping criterion of the main loop, a *PostProcessing* step is applied and illustrated in Fig. 1G and H. Any remaining active PVJs that were not connected in the main section of the method are handled in this step. First, we prune all extra branches of the tree. A branch is considered extra if it is not in a pathway that links an active PVJ to the root, as represented by the blue branches in Fig. 1G. Next, the LAT error tolerance constraint is dropped, and we attempt to connect all unconnected PVJs in $S_{a}$ using the pruned tree.

However, after this procedure, some PVJs may still be unconnected. This scenario could happen if $x_{PVJ}$ is already close to the current tree or in case of collisions. Therefore, we drop the distance criterion for these PVJs and attempt the connection again. Finally, if there are still unconnected PVJs, we connect them to the segment with the lowest LAT error among the $N_{a}$ closest segments, using a straight line. Therefore, we solve the minimization problem (5) by incrementally losing the restrictions $R_a$.

After this step, all the active PVJs that the user specified are connected, and the geometric and electric metrics are computed in this topology, which is referred to as the *minimum network*. More information and details regarding all the steps and parameters of the method are supplied in the Supplementary Material section A.7.
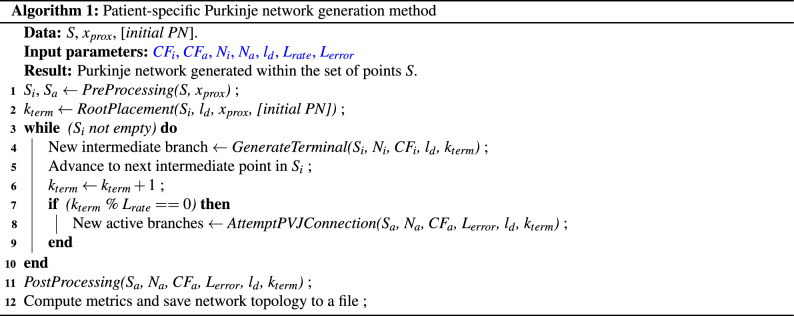

### Biventricular models

**Figure 2:**
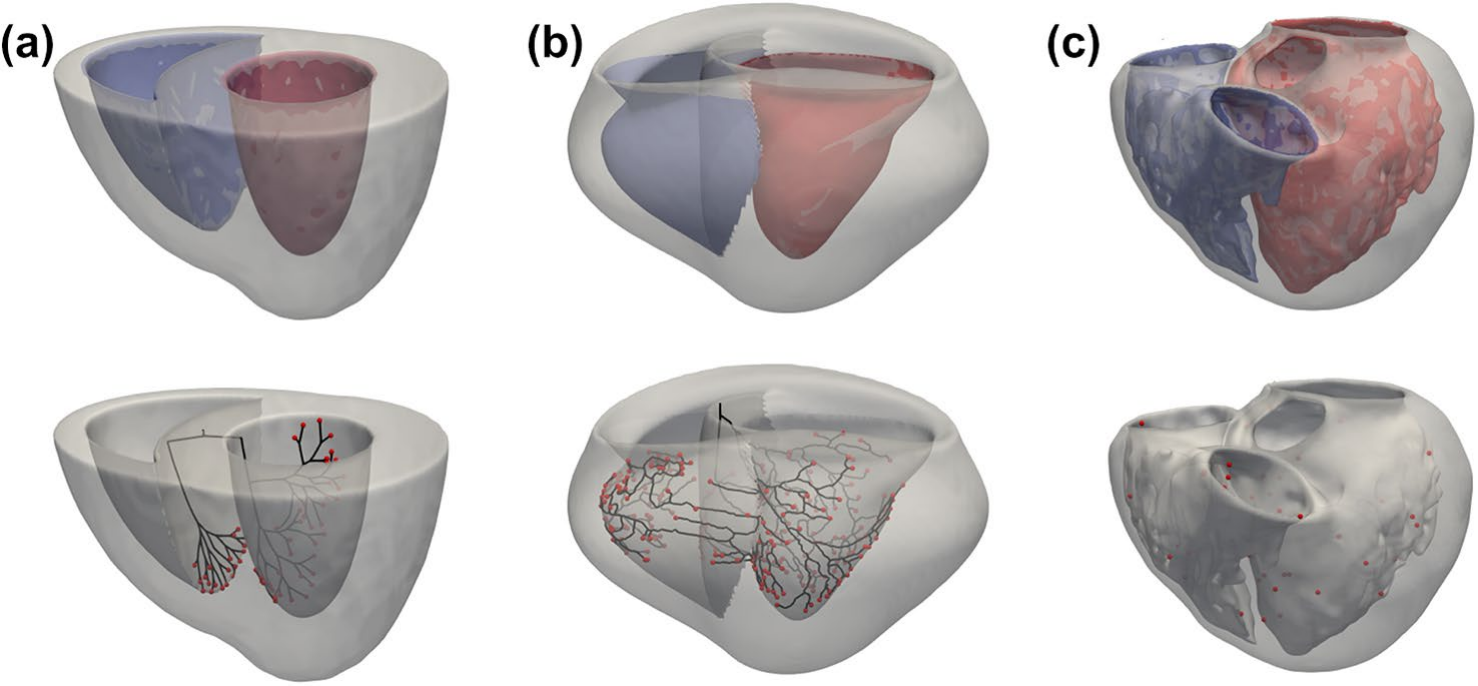
Biventricular meshes used during the experiments of the present work. In panel (**A**), the simplified mesh alongside the corresponding reference PN (black) and their active PVJs points (red). In panel (**B**), the canine mesh from^17^ is highlighted with the gold standard PN (black) and their active PVJs points (red). In panel (**C**), the patient-specific mesh from^28^ is depicted with its active PVJs points (red). The left ventricle is colored red in all the upper panels, while the right ventricle is blue.

Three different meshes were used to evaluate the robustness of our method. The first considers a simplified mesh that captures the main structures of the biventricular system. Secondly, a canine mesh is the target of the study. A human patient-specific mesh is utilized as the final experiment to evaluate our model. These meshes are shown in Fig. 2.

The simplified biventricular synthetic mesh was constructed via ellipsoids, for the left ventricle (LV) and right ventricle (RV) endocardium and epicardium. The main idea consists of subtracting the ellipsoids and cutting their surfaces along the base plane, as shown in Fig. 2A. Fiber orientation and transmural distance of the mesh were calculated using the Laplace-Dirichlet Rule-Based (LDRB) algorithm^29^. In addition, a simplified PN was generated using a fractal method^22^. This simplified PN was considered as our reference for comparisons. The branches of the reference PN activate the endocardium at the early region sites of the LV and RV as reported in^22,30^. For instance, these regions are close to the apex in the left and right ventricles and the posterior-basal region of the left ventricle. The locations of the PVJs and the His-bundle were manually selected and constructed to provide an activation pattern similar to what is observed physiologically^30^. All the terminals of the reference PN are considered active PVJs, representing a total of 43 and 20 PVJs in the LV and RV, respectively. Detailed information about the simplified mesh construction, as well as the parameters set used to generate the reference PN with the fractal method are described in the Supplementary Material, Section A.2.

A biventricular canine mesh whose PN was reconstructed from histological images^17^ and later processed into a graph representation^25^ was considered as our reference for comparison and can be visualized in Fig. 2B. In the previous work proposed by Ulysses et al.^25^ only the LV was considered in the experiments. In the present work, both ventricles are utilized to better evaluate the proposed method. Similar to the simplified mesh case, fiber orientation and transmural distance of the mesh were calculated using the LDRB algorithm^29^. Furthermore, since only the reference PNs of the LV and RV were available, we manually built the His-bundle structure. The provided data has 130 and 98 PVJs over the LV and RV, respectively.

A biventricular human patient-specific mesh utilized previously^18,28^ was used to further evaluate our novel method in a more realistic scenario, as illustrated in Fig. 2C. Along with the mesh, the locations and LATs from the active PVJs were estimated using an electroanatomical map (EAM) and contact-mapping catheter systems (CARTO3 ^TM^, Webster BioSense Inc.)^31,32^, with 31 and 16 active PVJ points on the LV and RV surfaces, respectively. To allow time for the Purkinje to grow branches and activate the estimated PVJs, a $40\textrm{ms}$ shift is introduced in their original LAT, i.e., after this shift, the earliest LAT is $t=40\textrm{ms}$. For this particular mesh, fiber orientation was already available. However, our studies did not consider any ischemic or fibrotic regions. In addition to the estimated PVJ sites, an extra set of points from the EAM located in healthy regions was selected to validate the patient-specific PN model. An essential aspect of the experiments using the patient-specific mesh is that we do not have a reference PN for comparison; only the early activation sites and some EAM points in the endocardium are available.

For each of these meshes, we randomly generated the sets *S*, which is the main input parameter of the method (see the previous section), by sub-sampling the set of points on the endocardial surfaces of the meshes.

### Numerical methods

#### Monodomain model

Cardiac myocytes are electrically coupled through gap junctions, a specialized set of proteins that allow the spreading of the electrical wave of excitation from one cell to another in the tissue. This phenomenon can be described by a reaction-diffusion equation, called the monodomain model^33^, which is given by

7 $$\begin{aligned} \beta C_m \frac{\partial V}{\partial t} + \beta I_{ion}(V,\vec {\eta })&= \nabla . (\sigma \nabla V) + I_{stim}, \end{aligned}$$

8 $$\begin{aligned} \frac{\partial \vec {\eta }}{\partial t}&= f(V,\vec {\eta }), \end{aligned}$$

where *V* is the transmembrane potential, $I_{ion}$ is the total ionic current that may also depend on gating variables $\vec {\eta }$, $\beta$ is the surface-volume ratio, $C_m$ is the membrane capacitance, $I_{stim}$ is the current due to an external stimulus, and $\sigma$ is the monodomain conductivity tensor. The model is further equipped with appropriate initial conditions and no-flux boundary conditions: $\sigma \nabla V. \vec {n} = 0$ on $\partial \Omega$, where $\vec {n}$ is the normal vector of the surface, $\partial \Omega$.

In the present work, when the monodomain model was applied, the Purkinje networks were solved using the one-dimensional form of the equation. On the other hand, for ventricular tissue modeling, the three-dimensional form of Eq. (7) was used with an anisotropic conductivity tensor. Regarding the cellular models used for the ionic current $I_{ion}$ term, the Trovato et al., model^7^ for human Purkinje cells and the recent version of the *ToRORd* model^34,35^ for human ventricular cells were applied to provide a sinus rhythm activation.

To numerically solve the monodomain equations and simulate the action potential propagation in both the Purkinje networks and the ventricular tissue, we used the *MonoAlg3D*, which is an efficient parallel cardiac solver^36^. This particular solver can handle non-uniform, non-conforming adaptive meshes and, most importantly, use the graphics processing unit to speed up the solution of both the diffusion and reaction terms associated with the model.

Regarding the parameters to enable the solution of the monodomain model, the surface-to-volume ratio was set to $\beta$ = 0.14 $\upmu$m and the tissue capacitance to $C_m = 100$ pF/$\upmu \textrm{m}^{2}$. A total simulation time of $t_{max} = 200$ ms was used and a time discretization $dt = 0.02$ ms was used to solve the associated partial differential equation (PDE). For the ordinary differential equations (ODEs) system related to the Purkinje and ventricular cellular models, a Rush-Larsen scheme with a fixed timestep of $dt=0.01$ ms was utilized. A space discretization of $400 \upmu$m was used for all the ventricular domains, while for the Purkinje network this value was set to $100\,\,\upmu$m. The conductivities from the ventricular domain were anisotropic with $\sigma _l=0.75$ S/m, $\sigma _t=0.225$ S/m and $\sigma _n=0.1125$ S/m, in the longitudinal, transverse and normal directions, respectively, resulting in CVs close to physiological values given by Durrer et al.^30^. For the Purkinje domain the conductivity was set to $\sigma _{purk} = 2.567$ S/m, with a CV approximately equal to 2 m/s. The stimulus protocol was a single pulse coming from the His bundle with the following parameters: $I_{amp}$ = 40 pA/pF, $\text {duration} = 2$ ms, $N_{cells} = 25$.

In addition, for the cellular dynamics, the *ToRORd* model for human ventricular cells^34,35^ and the Trovato et al. model for Purkinje cells^7^ were used mainly because these two cellular models are now considered the latest models in terms human cellular model available in the literature, presenting important advances over previous models like the classical *TT3* model^37^ and the O’Hara-Rudy model^38^ in several aspects. Similarly, the Trovato et al., Purkinje model provides several features that the Stewart et al., model^39^ was not able to capture. Moreover, to the best of our knowledge, there was no published work that coupled both models in a Purkinje-biventricular simulation.

To properly evaluate the activation time generated by the PNs, the monodomain equation was solved both for the Purkinje and ventricular domain, instead of the coupled Eikonal model seen in the previous work from^25^. This approach was used to capture essential features of propagation from Purkinje and ventricular cells accurately including at the PVJ sites, such as the characteristic delay that happens at these locations^10^, giving rise to a more realistic simulation in terms of cardiac electrophysiology. For a detailed description of how the monodomain model is solved for the Purkinje-coupled simulations check Supplementary Material section A.8.

The parameters of the cable equation used in the generation of the PNs were chosen by comparing the conduction velocity of Eq. (4) and that obtained using the monodomain equation on a 10 cm linear cable composed of cells modeled with the Purkinje human model^7^. Constraining the values to those observed in experiments^40^, we arrived at $G = 7.9\,\, \textrm{m}\Omega /\textrm{cm}$, $C_f = 3.4 \,\,\upmu \textrm{F}/\textrm{cm}^2$, $\tau _f = 0.1$ ms and $d=68.86\,\, \upmu \textrm{m}$.

#### Purkinje-Ventricular-Junctions

To model the Purkinje-Ventricular-Junctions sites, an additional passive current, $I_{PVJ}^{tiss}$, was included in the tissue cells that are coupled to the terminal cells of the Purkinje network:

9 $$\begin{aligned} I_{PVJ}^{tiss} = \sum _{i=0}^{N_{PVJ}} \dfrac{\left( V^{purk} - V_{i}^{tiss}\right) }{R_{PVJ}}, \end{aligned}$$

where $V^{purk}$ is the transmembrane potential of the terminal Purkinje cells, $V^{tiss}_{i}$ is the transmembrane potential of the ventricular cells *i* attached to the Purkinje cell, $R_{PVJ}$ is a fixed-resistance and $N_{PVJ}$ is the number of connecting ventricular cells. This additional current is included on the right-hand side of the linear system associated with the numerical discretization of the monodomain equation of the ventricular domain.

Similarly, for the Purkinje cells, a current, $I_{PVJ}^{purk} = -I_{PVJ}^{tiss}$, couples ventricular cells to a terminal Purkinje cell. Likewise, this is included on the right-hand side of the associated linear system of the Purkinje domain and is given by the following equation:

10 $$\begin{aligned} I_{PVJ}^{purk} = \sum _{i=0}^{N_{PVJ}} \dfrac{\left( V_{i}^{tiss} - V^{purk}\right) }{R_{PVJ}}. \end{aligned}$$

In Supplementary Fig. S2, we illustrate the Purkinje coupling model used in this work and how the terminal Purkinje cell is coupled using a fixed-resistance $R_{PVJ}$ to the nearest $N_{PVJ}$ ventricular cells.

#### Error analysis

To properly evaluate the geometrical features of our PN models, the mean and standard deviation of the branch size and angle of the bifurcations are computed together with the total number of branches and bifurcations.

To assess the activation accuracy of our model, the minimum/maximum LAT from the active PVJ sites were computed along with the maximum absolute error observed in these points in the generated PN. In addition, the root mean square error (RMSE) and relative root mean square error (RRMSE) between the LAT from the active PVJ sites of the reference points and the generated networks were calculated as follows:

11 $$\begin{aligned} \begin{aligned} RMSE&= \Bigg ( \frac{ \sum _{i=1}^{N} \left( \bar{y_i} - y_i \right) ^{2} }{ N } \Bigg )^{1/2}, \end{aligned} \quad \begin{aligned} RRMSE&= \Bigg ( \frac{ \sum _{i=1}^{N} \left( \bar{y_i} - y_i \right) ^{2} }{ \sum _{i=1}^{N} {(y_i)^{2}} } \Bigg )^{1/2}, \end{aligned} \end{aligned}$$

where $\bar{y_i}$ is the reference value, $y_i$ is the approximated value, and *N* is the number of samples. Finally, the percentage of the active PVJs within a specific range of error (i.e., 2 ms and 5 ms), given by the values $\varepsilon < 2$ ms and $\varepsilon < 5$ ms, was also considered.

## Results and discussion

### Sensitivity analysis of the input parameters

To evaluate the robustness of our method, we performed a sensitivity analysis over the input parameters. Our baseline simulation considered the generation of 10 PN trees for the simplified mesh with the following parameter set: $N_{i}=80$, $N_{a}=80$, $L_{rate}=25$, $l_{d}=10$ mm, $L_{error}=2$ ms. The RMSE error at the PVJ sites and the total execution time to generate the PNs were utilized to compare the different parameter combinations, as shown in Table 1.

**Table 1:** Sensitivity analysis of the input parameters for the baseline configuration of $N_{i}=80$, $N_{a}=80$, $L_{rate}=25$, $l_{d}=10$ mm, $L_{error}=2$ ms.

Parameters	Values	RMSE (ms)	Total execution time (min)
$N_{i}$	20	0.95 ± 0.31	1.81 ± 0.30
	400	0.95 ± 0.31	5.80 ± 0.54
$N_{a}$	20	1.20 ± 0.42	1.55 ± 0.12
	400	0.86 ± 0.22	6.68 ± 1.75
$L_{rate}$	10	0.95 ± 0.33	2.85 ± 0.55
	40	0.91 ± 0.32	2.36 ± 0.17
$l_{d}$	5 mm	1.10 ± 0.66	3.39 ± 0.34
	15 mm	1.18 ± 0.43	1.69 ± 0.15
$L_{error}$	1 ms	0.90 ± 0.33	2.54 ± 0.32
	5 ms	1.30 ± 1.06	2.56 ± 0.32
Baseline	–	${0.95} \pm {0.31}$	${2.50} \pm {0.31}$

The mean and standard deviation of the RMSE (milliseconds) at the PVJ sites and the total execution time (minutes) to generate the networks are listed.

Based on the results of Table 1, the number $N_{i}$ does not significantly affect the accuracy of the trees. On the other hand, large values for $N_i$ increase the execution time, since this implies more segments being evaluated by the geodesic pathway algorithm, which is computationally expensive. The results suggest we can obtain faster results without sacrificing accuracy by decreasing $N_{i}$.

Analyzing the other parameters, we could verify that the $L_{rate}$, $l_{d}$ and $L_{error}$ parameters do not affect substantially the results of activation accuracy and execution time.

Activation accuracy and execution time are most sensitive to the number $N_{a}$ of branches with lowest estimated LAT errors considered, as can be verified in Table 1. Unlike the case with $N_{i}$, the use of proxy of the LAT error to sort the candidate segments for a connection affects both accuracy and execution time. From the results, we see that decreasing the use of the proxy improves the trees electrically. In this regard, the $N_{a}$ parameter should be carefully adjusted to generate more precise PNs in a reasonable amount of time.

As a result of this initial analysis, we performed a new set of simulations varying $N_{a}$ to find the optimal value for activation accuracy and total computation time for this particular scenario. Our baseline configuration for this second analysis considers $N_{i}=20$, $N_{a}=80$, $L_{rate}=25$, $l_{d}=10$ mm, $L_{error}=2$ ms, and the results are shown in Table 2.

**Table 2:** Sensitivity analysis of the $N_{a}$ input parameter considering the baseline configuration.

Parameters	Values	RMSE (ms)	Total execution time (min)
$N_{a}$	80	0.95 ± 0.31	1.78 ± 0.28
	120	0.88 ± 0.30	2.39 ± 0.41
	160	0.88 ± 0.27	2.89 ± 0.60
	200	0.86 ± 0.22	3.39 ± 0.79
	240	0.86 ± 0.22	3.79 ± 1.00
	280	0.86 ± 0.22	4.33 ± 1.17
	320	0.86 ± 0.22	4.86 ± 1.38
	360	0.86 ± 0.22	5.45 ± 1.55
	400	0.86 ± 0.22	5.99 ± 1.75
Baseline	–	${0.95} \pm {0.31}$	${1.78} \pm {0.28}$

The mean and standard deviation of the RMSE (milliseconds) at the PVJ sites and the total execution time (minutes) to generate the networks are listed.

When $N_{a}$ is greater than 120, the RMSE converges to a value around 0.86 ms. Within this context, we conclude that it is not necessary to keep increasing this parameter to improve the activation accuracy of the PNs. This finding confirms that, although not entirely accurate, sorting the feasible segments using a proxy is beneficial.

### Biventricular simulations demonstrate that multiple Purkinje networks can share similar properties

The method proposed in this work was evaluated using three different biventricular meshes of increasing complexity. For each mesh, we randomly generated 100 sets *S*, which is the main input parameter of the method, by sub-sampling the set of points on the endocardial surfaces of the meshes. A total of 200 PNs were generated for each mesh, including 100 PNs for the LV and the remainder for the RV. RMSE was calculated for each PN by comparing the LATs at the reference active PVJ sites. The PN samples were sorted by their RMSE values, and the ten best PNs for each ventricle region were used to compute the metrics and statistics. Based on the results of the sensitivity analysis, the parameters of the performed simulations were set to $N_{i}=20$, $L_{rate}=25$, $L_{error}=2$ ms and $d=69\upmu \textrm{m}$, giving a CV of approximately 2 m/s when using the cable Eq. (4) with Purkinje parameters. For the simplified and canine meshes, $l_{d}=10$ mm and $N_{a}$ was set to 120 for both ventricles. For the patient-specific mesh, $l_{d}=30$ mm and $N_{a}$ was set to 200 for both ventricles. The choice regarding the $l_{d}$ parameter reflects that the patient-specific mesh has a larger volume than the simplified and canine meshes, while for the $N_{a}$ parameter the larger value improved the accuracy of the generated PNs. All simulations were performed without any restriction regarding the bifurcation angle and segment length. The geometrical and activation features of all PNs were computed considering only the minimum networks (after the *PostProcessing* procedure). Consequently, all terminals of the resultant PNs were connected to active PVJs. More details regarding the geometrical and activation results from the ten best PNs for each mesh are presented in the tables within the Supplementary Material, Sections A.4 and A.5.

**Figure 3:**
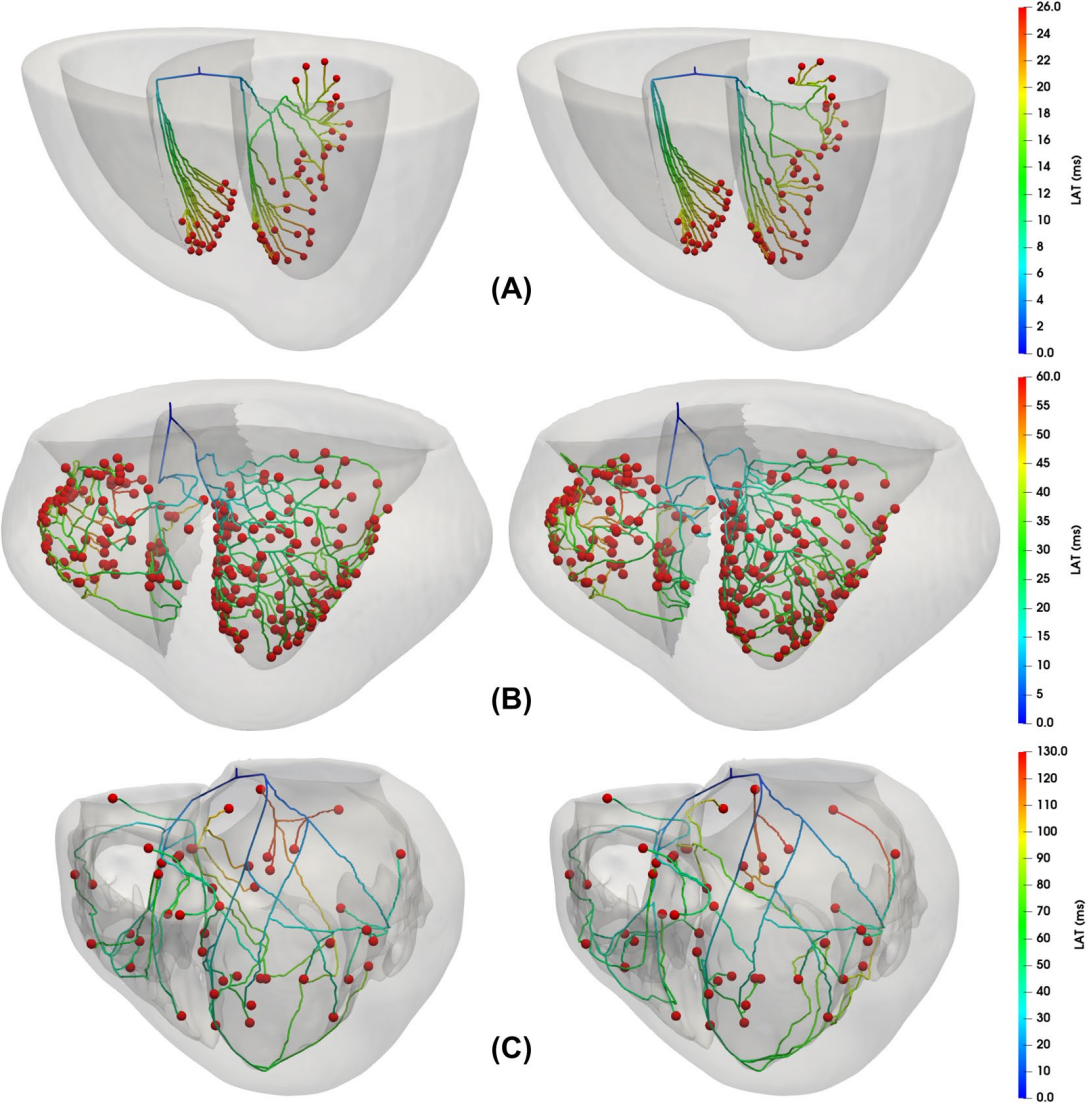
Comparison of the PN morphologies generated by the method for each biventricular mesh. The figure depicts the best (left) and worst (right) networks regarding their LATs at the PVJ sites for the ten PNs of the comparison set.

Figure 3 presents the results for the three different meshes. In addition, we present for each mesh the best (left) and worst (right) networks with respect to their LATs at the PVJ sites for the ten PNs of the comparison set. We note that, for small changes to the input *S*, the method generated different PN morphologies with similar LATs at the active PVJ sites. For this reason, even for the same set of PVJs, it is possible to have multiple PN solutions that share LATs close to the target values. When the best and worst PNs were compared, as shown in Fig. 3, the same PVJ site could be activated within the LAT error tolerance through different pathway combinations.

### Variability in the geometrical features of the generated Purkinje networks preserve the matching of the activation features

**Figure 4:**
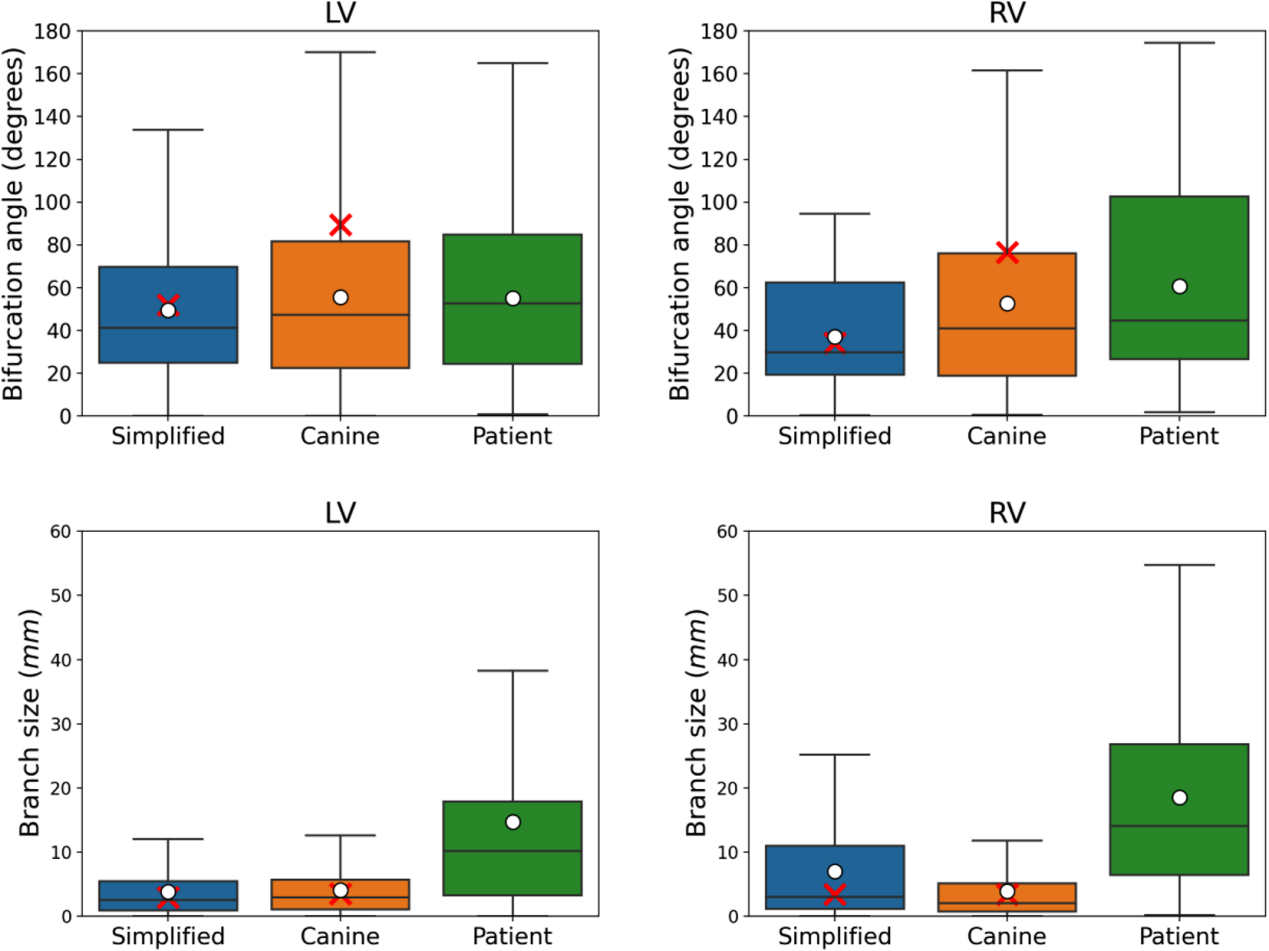
Geometrical results from both ventricles of the ten best PNs generated for each biventricular mesh. The boxplot for the bifurcation angle, given in degrees, is illustrated in the top panel, while the boxplot for the branch size, given in millimeters, is in the bottom panel. The red cross denotes the mean reference values for the gold standard PNs of the simplified and canine meshes.

**Figure 5:**
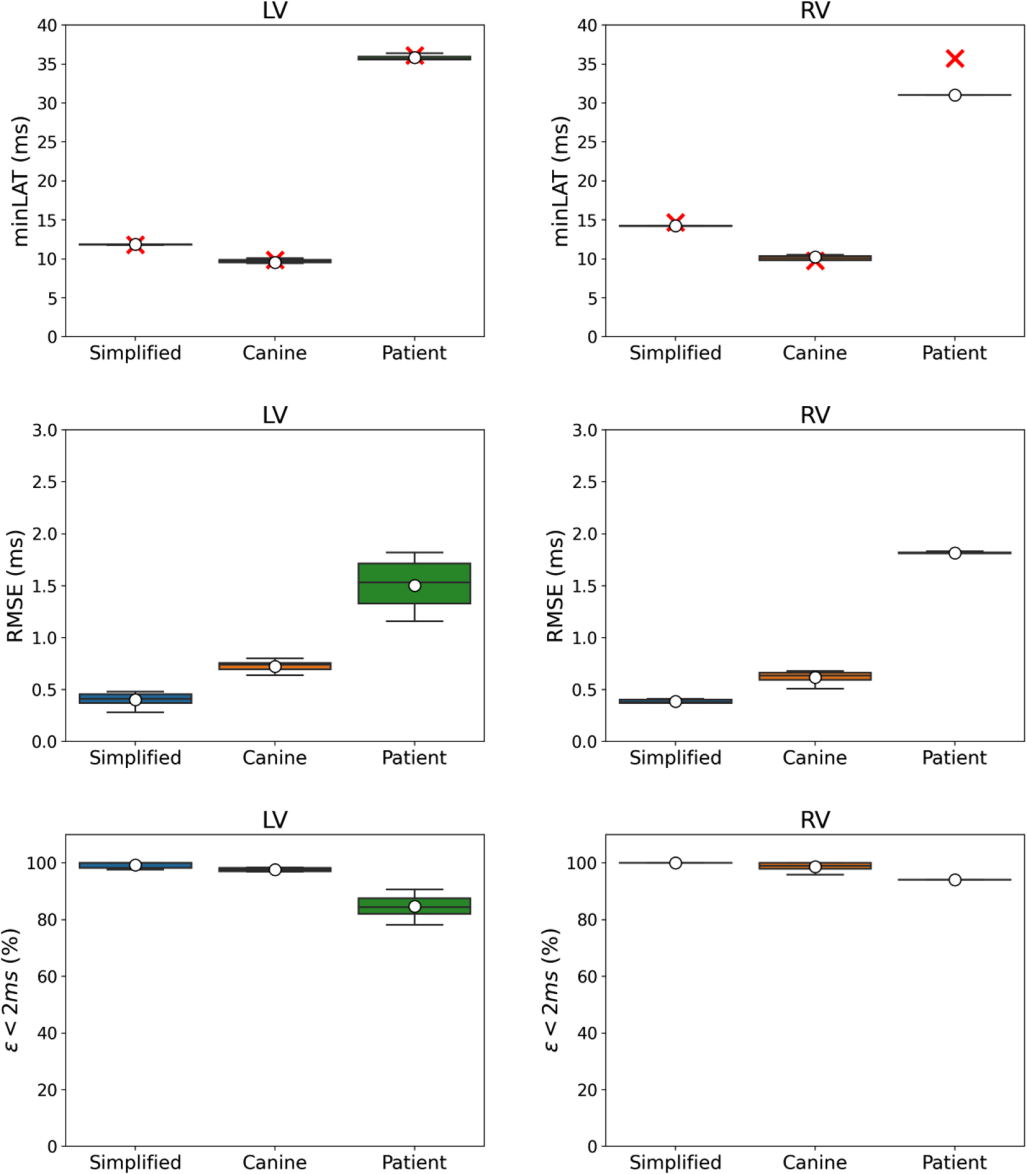
Activation results from both ventricles for the ten best PNs generated for each biventricular mesh. The minimum LAT, RMSE error, and $\varepsilon <2$ ms values were recorded at the active PVJ sites. The boxplot for the minimum LAT is presented in the top panel, where the red cross denotes the mean reference values for all the meshes. The boxplot for the RMSE error in the middle panel was calculated using only the PNs at the PVJ sites. Finally, in the bottom panel, we present the percentage of PVJs connected within a 2 ms tolerance.

Analyzing the geometrical results given in Fig. 4, the proposed method shows good accuracy compared to the available reference values. For instance, the simplified mesh PNs present a bifurcation angle with a mean value of approximately $49.51^{\circ }$ and $37.21^{\circ }$ for the LV and RV trees, respectively. Considering the same ventricular regions, these values are close to the target values of approximately $52.02^{\circ }$ and $34.23^{\circ }$. For the canine mesh, the generated PNs presented more variability in this feature with a mean value around $55.56^{\circ }$ and $52.73^{\circ }$ for the LV and RV trees, respectively, which are far from the reference values of approximately $89.48^{\circ }$ and $76.58^{\circ }$. In the case of branch size, the mean value for the simplified mesh is around 4.40 mm and 6.66 mm for the LV and RV trees, respectively, while the target values are close to 2.93 mm and 3.41 mm. For the canine mesh, the generated PNs returned a mean value of approximately 4.18 mm and 4.07 mm, while the reference values are between 3.56 and 3.43 mm, respectively. The results for the patient-specific mesh could not be compared to any reference value since we do not have a gold standard PN for this scenario.

Furthermore, the bifurcation angle and branch size values of the patient-specific mesh varied more than the simplified and canine meshes. For instance, the mean value of the bifurcation angle is approximately $55.25^{\circ }$ and $60.66^{\circ }$ for the LV and RV trees, respectively, while for the branch size the mean value is around 16.48 mm and 18.70 mm for the same ventricular regions. This behavior may be related to the patient-specific mesh having the largest volume, leading to longer branches and angles to connect the active PVJs within the LAT error tolerance. In addition, the high variability that can be observed in the results of Fig. 4 can be explained due to the fact that no restriction was imposed on the geometrical features of the PN.

As can be seen in Fig. 5, the activation results for the PNs generated for the simplified and canine are in almost perfect agreement with the reference values for minimum LAT. Moreover, the PNs of these two meshes do not present a high variability in this metric. On the other hand, the patient-specific mesh shows a difference of approximately 5 ms in the minimum LAT of the RV region. This phenomenon indicates that the PVJ with the lowest LAT was not connected during the main loop of the method.

From the RMSE results in Fig. 5 we observe that the error increases as the mesh complexity increases. For instance, the RMSE was below 0.5 ms for both ventricles of the simplified mesh and below 1 ms for both ventricles of the canine mesh. For the patient-specific mesh, the RMSE was below 2 ms. The results for the LV varied more than those for the RV.

Finally, the fraction of PVJs that were connected within a LAT error tolerance of 2 ms ($\varepsilon <2$ ms metric in Fig. 5) was above $75\%$ in all scenarios. Within this context, the method was able to generate PNs with reasonable accuracy in terms of LAT.

### Mesh complexity affects the method computational performance

In addition to the geometrical and activation results, we measured important features related to the computational performance of the method. The execution time spent on each section of the program, the percentage of time required for the geodesic pathway calculus and the percentage of PVJs connected in each step were calculated and are presented in Tables 3 and 4 . The method was divided into four phases: the main loop and three steps of the *PostProcessing* subroutine, which correspond to the LAT error tolerance and distance criterion removal (geodesic and straight line connections).

The results in Table 3 show that the main loop is responsible for the majority of the execution time with a percentage above $90 \%$ in almost all scenarios. In particular, for the PNs generated in the LV of the simplified mesh, the percentage is around $79 \%$. This behavior can be explained due to the fact that a certain number of PVJs could not be connected due to branch collisions to intermediate branches that were still present in the tree before the pruning that occurred at the beginning of the *PostProcessing* subroutine. A further reason is related to PVJs being already too close to the tree, which means that these points could be connected only by removing the distance criterion constraint, as confirmed in Table 3 by large computation times for Phases II, III + IV, with percentages close to $7 \%$ and $13 \%$, respectively. Table 4 also verifies that almost half of the PVJs were connected during these three phases of the *PostProcessing* function for the simplified mesh.

Table 3 also shows that the computation time increased with the mesh complexity. The mean execution time to generate a PN for the simplified mesh was around 2 min in both ventricles. For the canine mesh, this time was approximately 3 min for the LV and 5 min for the RV. For the patient-specific mesh, the time was close to 19 min for the LV and 7 min for the RV. This increase in computation time in the LV trees of the patient-specific mesh can be explained by two main factors. First, the patient-specific mesh has several physiological features that do not appear in the other two meshes, including the papillary muscles and a very irregular endocardial surface with tendons, which increased the total number of triangular elements necessary to represent these particularities. After measuring the computation times of the different subroutines of the method, we observed that the computation of the geodesic pathways was responsible for $75\%$ of the total execution time. The complexity and computation time of calling the geodesic pathway procedure increased in proportional to the number of elements of the covered surface. In addition, to improve the accuracy of the generated PNs for the patient-specific mesh we increased the $N_{a}$ parameter, which, as presented in the sensitivity analysis section, also increased the computation time of the method.

Table 4 presents the percentage of PVJs connected during each phase of the method. By examining Phases III and IV, we verified how many PVJs were connected by either a geodesic pathway or straight lines after the distance criterion removal. From these results, when considering the simplified mesh, from the $29.53 \%$ PVJs of the LV trees connected during Phases III and IV, $23.49 \%$ are connected using a geodesic pathways, while $6.05 \%$ of the PVJs are linked using a straight lines in Phase IV. For the RV trees, from the total of $19 \%$ PVJs connected during the third and fourth phases, $5.50 \%$ of the PVJs were connected via geodesic and $13.50 \%$ used straight lines in the fourth phase. For the canine mesh, a total $10.69 \%$ of the PVJs located in the LV region were connected after eliminating the distance criterion, whereas $4.23 \%$ were linked using geodesic and $6.46 \%$ with a straight line. In the RV region, the same percentages from the total of $26.33 \%$ PVJs connected after the distance criterion was dropped, $10.92 \%$ were linked using geodesic and $15.41 \%$ with straight lines. Finally, for the patient-specific mesh, from the total of $26.13 \%$ PVJs connected during Phases III and IV in the LV trees, $17.42 \%$ were connected by geodesic pathways and $8.71 \%$ using straight lines. For the RV trees, all $16.88 \%$ of PVJs connected in the third phase of the method were linked by geodesic pathways. Within this context, we can conclude that only a small fraction of the PVJs were forced to connect using straight lines during the last step of the method, as represented by Phase IV in the tables.

**Table 3:** Summary of the mean execution time for each phase of the method for all three biventricular meshes considering the time for the ten best PNs.

Mesh	Region	Phase I	Phase II	Phase III + IV	Total
		(min/%)	(min/%)	(min/%)	(min/%)
Simplified	LV	1.65/79.33	0.16/7.57	0.27/13.06	2.08/100
	RV	2.25/95.79	0.00/0.01	0.10/4.16	2.35/100
Canine	LV	2.87/94.47	0.03/0.95	0.14/4.56	3.04/100
	RV	4.23/90.94	0.10/2.16	0.32/6.88	4.65/100
Patient-specific	LV	17.77/92.58	0.35/1.81	1.08/5.61	19.20/100
	RV	6.42/92.87	0.17/2.42	0.33/4.70	6.92/100

Phase I is the main loop, Phase II is the first *PostProcessing* subroutine which drops the LAT error tolerance, and Phases III+IV are the subsequent procedures that eliminate the distance criterion to connect the remaining PVJs.

**Table 4:** Summary of the percentage of active PVJs connected on each phase of the method for all three biventricular meshes considering the mean values from the ten best PNs.

Mesh	Region	Phase I (%)	Phase II (%)	Phase III (%)	Phase IV (%)
Simplified	LV	54.19	16.28	23.49	6.05
	RV	81.00	0.00	5.50	13.50
Canine	LV	88.00	1.31	4.23	6.46
	RV	67.14	6.53	10.92	15.41
Patient-specific	LV	66.13	7.74	17.42	8.71
	RV	75.62	7.50	16.88	0.00

Phase I is the main loop, Phase II is the first *PostProcessing* subroutine which drops the LAT error tolerance, Phase III is the subsequent procedure that eliminates the distance criterion to connect the remaining PVJs but which utilizes a geodesic path to link the points and Phase IV is when the connection of the remaining PVJs after dropping the distance criterion occurs using a straight line.

### The generated Purkinje networks enables matching the reference LAT in monodomain simulations

To assess the generated PNs in 3D cardiac simulations, we simulate the activation of the ten best PNs for each mesh using a high-performance monodomain solver^36^ with parameters as previously described in the Methods section.

**Figure 6:**
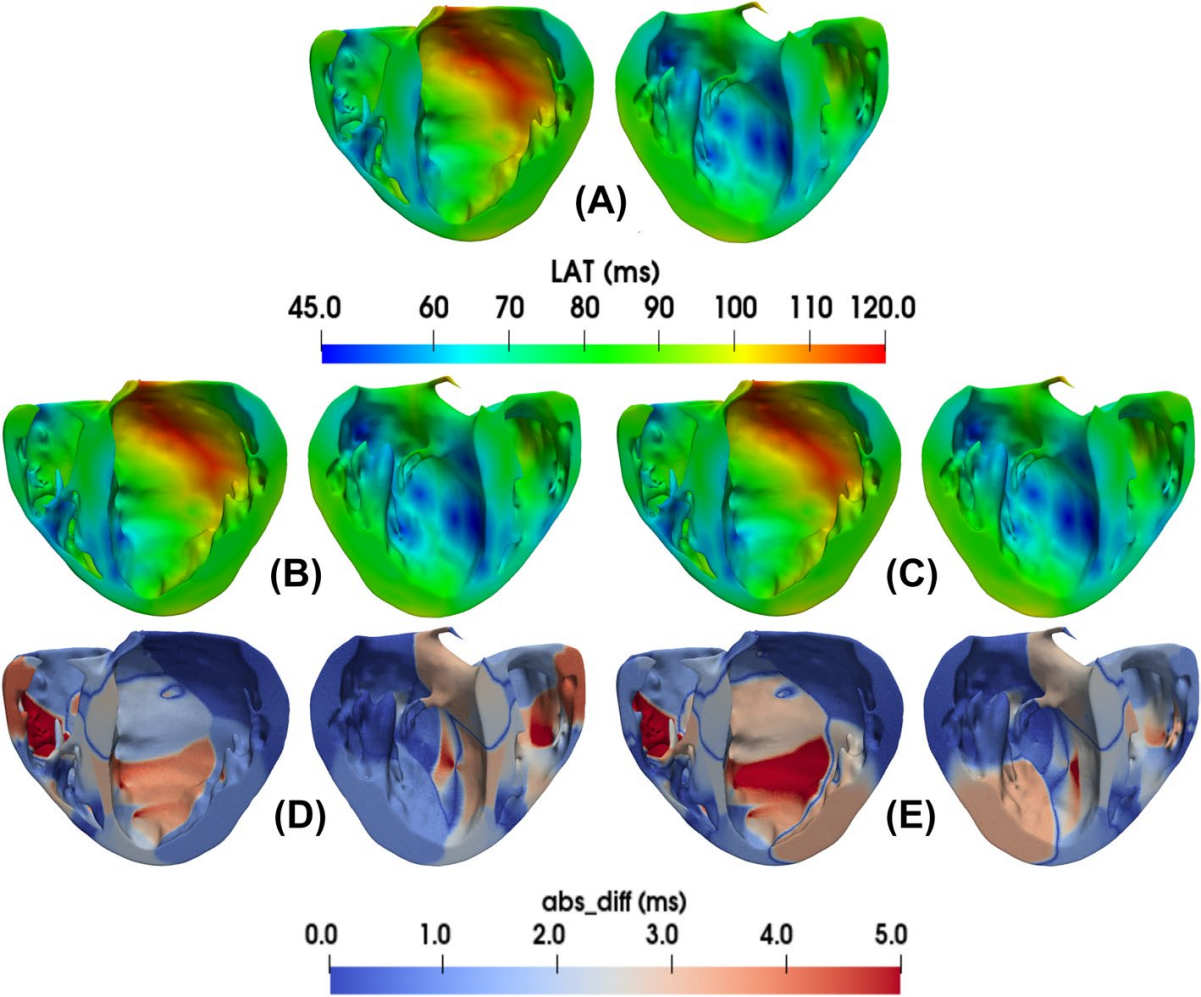
Results for the LAT maps for a coupled monodomain simulation using the patient-specific mesh. First, in the top panel (**A**), the active PVJs were activated following the LAT provided by the CARTO3 ^TM^ points. Next, in the middle panels (**B**) and (**C**), the LAT maps of the best and worst PNs generated by our method are depicted, respectively. Finally, in the bottom panels (**D**) and (**E**), the absolute LAT difference maps for the best and worst PNs, respectively, are shown.

For the patient-specific mesh, as depicted in Fig. 6, the ventricular activation can be quite complex and variable depending on the PN that is coupled to the tissue. The results for this case also show that at specific PVJ sites, the PN deviates from the reference value, especially near the regions around the papillary muscles of the RV. This behavior indicates that even with RMSE error below 3 ms, there are still regions with a lower LAT accuracy due to branches that were forced to connect during the final phases of the method. The LAT and absolute difference maps associated with the best and worst PN for the simplified and canine meshes can be analyzed in the Supplementary Material Section A.6.

To further investigate the differences in LATs presented in Fig. 6, we performed calculations to determine the absolute LAT difference between the LAT maps of the best and worst PNs using the patient-specific mesh (refer to Supplementary Material Section A.6.3). The primary objective of this experiment was to confirm whether the observed variations in LATs were primarily attributable to the distinct topologies of the PNs.

From Supplementary Material Fig. S6, it is evident that there are notable differences in the activation of the LV between the two networks, particularly in the apex region, where the absolute differences are between $3\textrm{ms}$ and $4\textrm{ms}$. Furthermore, in this area, we can observe distinct characteristics of the two PNs. It is important to acknowledge that, although numerical errors arise from spatial and temporal discretization, both simulations employed identical meshes and time steps in our experiments.

Therefore, this analysis provides support for the notion that the differing PNs are the primary cause of the observed disparities in LATs, particularly regarding the locations of the potential PVJ sites.

**Figure 7:**
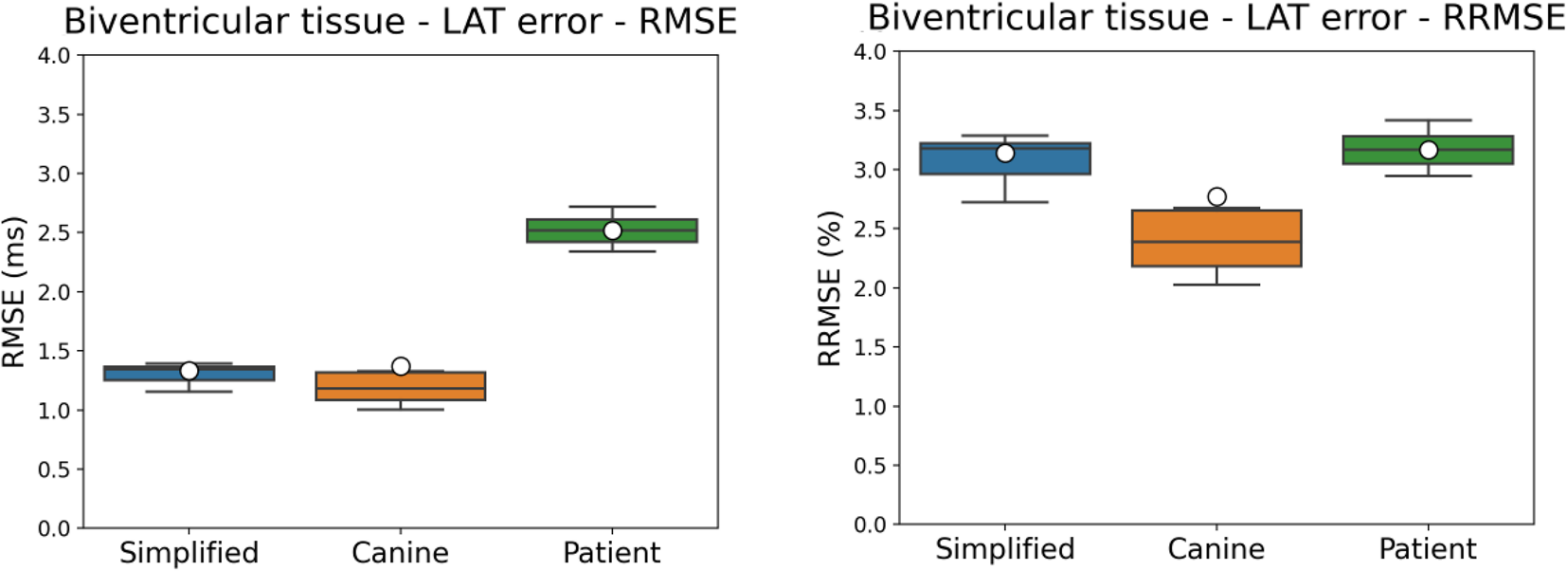
Results for the RMSE and RRMSE errors from the LAT maps of the biventricular tissues from the monodomain simulations. The RMSE and RRMSE errors were calculated for all the tissue cells by taking the difference between the LATs given by the reference times and the LATs generated by the ten best PNs for each mesh.

Figure 7 presents the RMSE and RRMSE errors from the LAT maps of biventricular monodomain simulations resultant from the ten best PNs for each mesh. For the simplified mesh, the RMSE and RRMSE values were around 1.4 ms and $3.2\%$, respectively. The Canine mesh generated lower RMSE and RRMSE values of around 1.3 ms and $2.5\%$, respectively, compared to the simplified mesh. For the patient-specific mesh, the RMSE and RRMSE values were around 2.5 ms and $3.1\%$, respectively. In general, these results indicate that the method is able to generate PNs with good activation accuracy.

The reported total execution times for constructing the patient-specific geometries demonstrate the method’s effectiveness in generating the PN in less than 20 min. This achievement is particularly impressive when compared to the time a specialist would typically spend on the initial manual steps involved in defining the PN, such as data importation and seed selection. Moreover, when considering the time required for generating ventricular geometry or simulating propagation, it becomes evident that the method remains highly efficient. It is worth noting that these results hold true even when considering state-of-the-art implementations for automatic segmentation, which can take in general $5-5.5\,\,\textrm{h}$ to prepare an optimal biventricular mesh from cine magnetic resonance slices^41^ or parallel simulation using modern GPUs^36^, that for the monodomain simulations done in this work took on average $36\,\,\textrm{min}$, $69\,\,\textrm{min}$ and $97\,\,\textrm{min}$ to execute for the Simplified, Canine and Patient-specific meshes respectively. These observations further highlight the practicability of our novel method.

### Electroanatomical map validation

**Figure 8:**
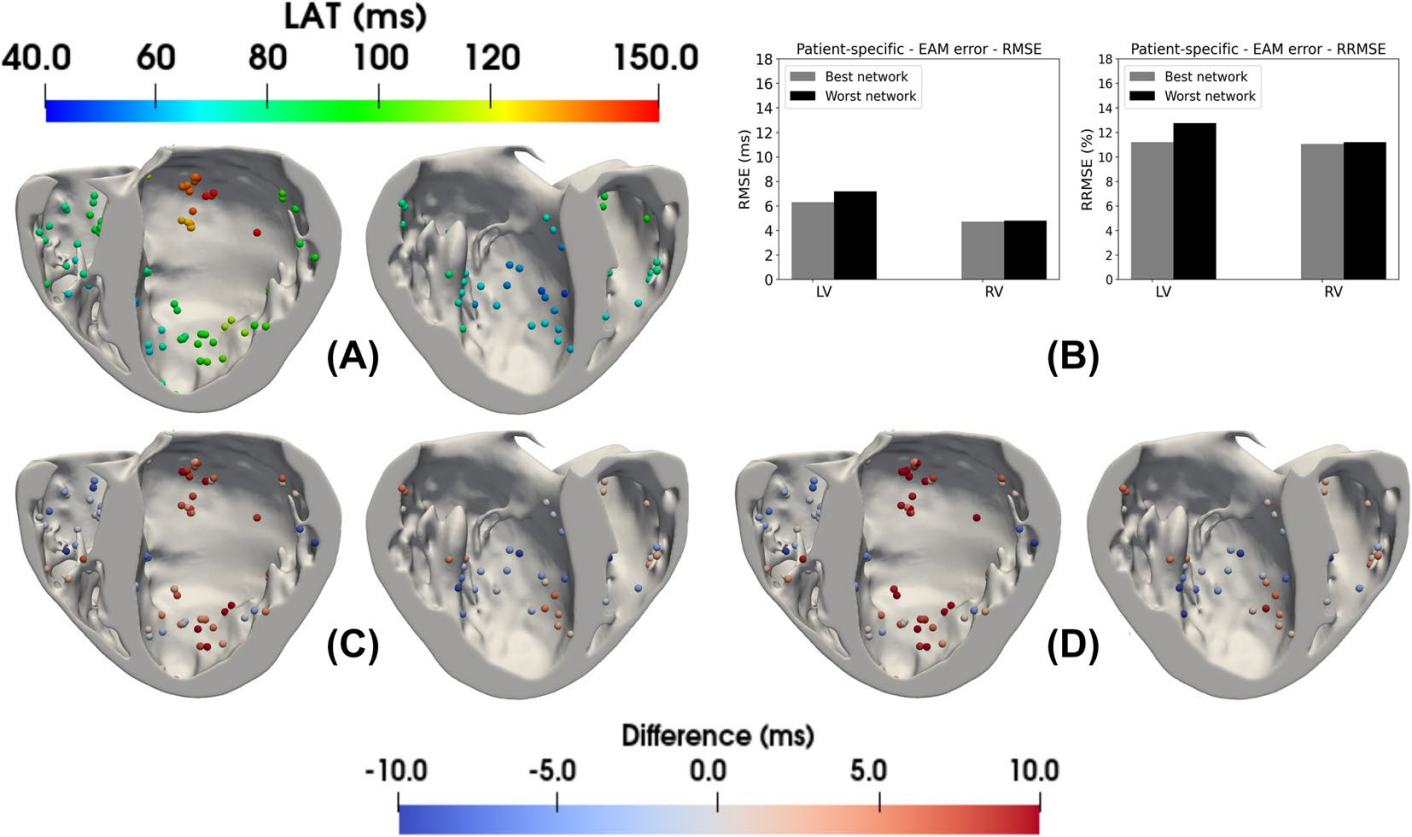
Overview of the EAM points selected for LAT validation is shown in panel (**A**). The results for the RMSE and RRMSE errors from the EAM points selected in the patient-specific LV and RV endocardial surfaces are shown in panel (**B**), where the errors were calculated for all the EAM points by taking the difference between the LATs given by the reference times and the LAT values from the closest tissue cell generated by a monodomain simulation with the best and worst PNs. In panels (**C**) and (**D**), the LAT differences at the EAM points for the best/worst PNs are illustrated, respectively.

As a final validation experiment, a subset of the original EAM points used to estimate the active PVJs in the patient-specific mesh was selected to compare the errors of the best and worst generated networks. Only EAM points within healthy regions of the endocardial surface in the LV and RV were considered. The points inside or near the damaged tissue that could be affected by fibrosis were eliminated, so that 100 and 47 EAM points were selected in the LV and RV, respectively.

3D monodomain simulations were performed considering our method’s best and worst generated PNs. Tissue conductivity was manually calibrated to reproduce a tissue LAT close to the ones provided by the EAM points. As shown in Fig. 8, the RMSE values were around 6.3 ms and 7.2 ms for the best and worst PNs at the EAM points located in the LV, respectively. For the points within the RV, the RMSE was around 4.8 ms in both PNs. The RRMSE values for the LV points were around $11.2\%$ and $12.7\%$ for the best and worst PNs, respectively, while for the RV the values were about $11.1\%$ in both PNs. The LV networks had the highest errors, indicating the complexity of matching activations around this region. In addition, another issue that might have caused an increase in the errors in both biventricular areas is associated with the manual adjustment of the tissue conductivity, which could result in a difference in conduction velocity in these regions.

## Limitations

Our simulations consider that the conduction velocity across the PN is constant to avoid an extra layer of complexity in the generation method. However, it is known that at bifurcations, there is a “push and pull” effect^42,43^ which decreases the conduction velocity. We have also considered the diameter of the Purkinje branches to be constant. However, physiological imaging studies have observed variations in the diameter of the structure depending on the location^19^. To address these limitations and improve the generation method, we propose, in the near future, incorporating a dynamic adjustment of the diameter of the Purkinje branches. This adjustment would account for the observed variations in diameter and allow for different conduction velocities along the network. By introducing this dynamic feature, we can enhance the physiological relevance and accuracy of our simulations.

In addition, based on the results it was noticed that the PVJ characteristic delay influences the activation of the ventricles. In the near future, we plan to take this delay into account in the active cost function by combining a data-based model, trained by monodomain simulations, and the cable equation.

In the present study, we have solely focused on functional PVJs, also known as active PVJs. This choice stems from the limited knowledge regarding both the density and activity of the junctions^44^. It is worth noting that several factors can influence the activity of a specific PVJ, including source-sink mismatch, electrotonic effects, and cellular dynamics^8,31,44,45^. Furthermore, emerging evidence suggests that non-functional PVJs can exhibit both pro-arrhythmic and anti-arrhythmic properties^44^, as they may serve as sites for re-entrant circuits or only become activated during network failures.

To advance our model and extend its applicability, it would be valuable to explore other techniques that incorporate non-functional PVJs as additional input parameters. This expansion would augment the range of applications for our novel method, providing a more comprehensive understanding of the complex behavior of the cardiac system.

In this study, our monodomain simulations are focused on the activation of PVJs originating from a single pulse initiated at the His-bundle. The primary objective was to match the LATs of the PVJ points and validate our Purkinje generation method. However, we acknowledge that the density and coverage of PVJs within a specific PN can influence its propensity to generate reentry pathways, potentially leading to ventricular fibrillation^46,47^.

To address this aspect comprehensively in future work, uncertainty quantification studies can be employed. By generating multiple PNs capable of reproducing similar LATs, these studies can assess the probability of PNs with reentry pathways for individual patients. Such investigations would enhance our understanding of the relationship between PVJ characteristics, PN topology, and the risk of ventricular arrhythmia, contributing to personalized assessments and interventions in clinical settings.

## Conclusion

Currently, implementing models for patient-specific PNs which combine geometric and activation precision are essential for more accurate development of cardiac electrical simulations. In addition, the recent increase in the development of cardiac digital twin (CDT) models makes the usage of Purkinje generation methods an essential step towards more realistic simulations. Furthermore, to study cardiac pathologies such as arrhythmias, models that consider PNs have substantial importance since this structure is known to be a source of ventricular fibrillation^48^ and its presence may influence behavior of these states^49,50^.

In this work, we presented a new method for the automatic construction of the PN to be used in cardiac electrical simulations. The method is an extension of our previous work, which is based on optimization principles. The method uses a new cost function that relies on electrical principles using the classic cable equation and targets LATs of a given set of active PVJs. We demonstrated the capabilities of the proposed method with the generation of different PNs for three biventricular meshes of increasing complexity. In addition, the electrical activity of the best PNs generated by our method was evaluated on a biventricular Purkinje-coupled monodomain simulation using the latest human ventricular/Purkinje cellular models and a high-performance GPU-accelerated solver.

Our results, both in terms of activation times and geometric features, indicate that the novel method can capture key aspects of the PNs (bifurcation angles, branch sizes, and LATs at the active PVJs sites) while preserving the structure within the endocardial surfaces, even near complex regions such as the papillary muscles. Furthermore, the method is flexible and scalable by allowing PNs generated by other methods to be used as initial roots. As a result, our method can further extend these initial networks to satisfy desired objectives, such as increasing the PN endocardial coverage or linking additional PVJs.

In the future, the applicability and performance of the method could be further improved and investigated. For instance, the use of additional electrical information, such as ECG readings, could be used to further constrain PN generation. The execution time of the method could be reduced by using parallel programming. Moreover, uncertainty quantification studies may provide new insights into improving our models and methods.

Finally, the novel method provides a new alternative for generating Purkinje networks for patient-specific heart models with controlled morphological metrics and specified local activation times at the Purkinje-ventricular junctions.

## Supplementary Information

Supplementary Information.

## Data Availability

The datasets generated during and/or analysed during the current study are available from the corresponding author on reasonable request. The source code for the novel patient-specific method for generating Purkinje networks presented in this work is available at https://github.com/bergolho/Shocker. Monodomain simulations were performed with the GPU-based MonoAlg3D software, available at https://github.com/rsachetto/MonoAlg3D_C.

## References

[1] Park DS, Fishman GI (2017). Development and function of the cardiac conduction system in health and disease. J. Cardiovasc. Dev. Dis..

[2] Haïssaguerre M (2002). Mapping and ablation of idiopathic ventricular fibrillation. Circulation.

[3] Haissaguerre M, Vigmond E, Stuyvers B, Hocini M, Bernus O (2016). Ventricular arrhythmias and the His-Purkinje system. Nat. Rev. Cardiol..

[4] Haissaguerre M (2019). Idiopathic ventricular fibrillation with repetitive activity inducible within the distal Purkinje system. Heart Rhythm.

[5] Imanishi R (2006). Prognostic significance of incident complete left bundle branch block observed over a 40-year period. Am. J. Cardiol..

[6] Walton RD (2014). Influence of the Purkinje-muscle junction on transmural repolarization heterogeneity. Cardiovasc. Res..

[7] Trovato C (2020). Human Purkinje in silico model enables mechanistic investigations into automaticity and pro-arrhythmic abnormalities. J. Mol. Cell. Cardiol..

[8] Boyden PA, Hirose M, Dun W (2010). Cardiac Purkinje cells. Heart Rhythm.

[9] Mendez C, Mueller WJ, Merideth J, Moe GK (1969). Interaction of transmembrane potentials in canine Purkinje fibers and at Purkinje fiber-muscle junctions. Circul. Res..

[10] Wiedmann RT, Tan RC, Joyner RW (1996). Discontinuous conduction at Purkinje-ventricular muscle junction. Am. J. Physiol.-Heart and Circul. Physiol..

[11] Li, J., Zhang, H. & Boyett, M. Numerical analysis of conduction of the action potential across the Purkinje fibre-ventricular muscle junction. In *2016 Computing in Cardiology Conference (CinC)* 265–268 (IEEE, 2016).

[12] Gillette K (2021). Automated framework for the inclusion of a His-Purkinje system in cardiac digital twins of ventricular electrophysiology. Ann. Biomed. Eng..

[13] Vergara C (2014). Patient-specific generation of the Purkinje network driven by clinical measurements of a normal propagation. Med. Biol. Eng. Comput..

[14] Palamara S (2014). Computational generation of the Purkinje network driven by clinical measurements: The case of pathological propagations. Int. J. Numer. Methods Biomed. Eng..

[15] Krishnamoorthi S (2014). Simulation methods and validation criteria for modeling cardiac ventricular electrophysiology. PLoS ONE.

[16] Stephenson RS (2017). High resolution 3-dimensional imaging of the human cardiac conduction system from microanatomy to mathematical modeling. Sci. Rep..

[17] Liu BR, Cherry EM (2015). Image-based structural modeling of the cardiac Purkinje network. BioMed Res. Int..

[18] Miralles, F. B. *Inverse estimation of the cardiac Purkinje system from electroanatomical maps*. Ph.D. thesis, Universitat de València (2020).

[19] Ono N (2009). Morphological varieties of the Purkinje fiber network in mammalian hearts, as revealed by light and electron microscopy. Arch. Histol. Cytol..

[20] Ijiri T (2008). A procedural method for modeling the Purkinje fibers of the heart. J. Physiol. Sci..

[21] Sebastian R, Zimmerman V, Romero D, Sanchez-Quintana D, Frangi AF (2012). Characterization and modeling of the peripheral cardiac conduction system. IEEE Trans. Med. Imaging.

[22] Costabal FS, Hurtado DE, Kuhl E (2016). Generating Purkinje networks in the human heart. J. Biomech..

[23] Bordas R (2011). Rabbit-specific ventricular model of cardiac electrophysiological function including specialized conduction system. Prog. Biophys. Mol. Biol..

[24] Barber F (2021). Estimation of personalized minimal Purkinje systems from human electro-anatomical maps. IEEE Trans. Med. Imaging.

[25] Ulysses JN (2018). An optimization-based algorithm for the construction of cardiac Purkinje network models. IEEE Trans. Biomed. Eng..

[26] Schreiner W, Buxbaum PF (1993). Computer-optimization of vascular trees. IEEE Trans. Biomed. Eng..

[27] Keener JP, Sneyd J (1998). Mathematical Physiology.

[28] Lopez-Perez A (2019). Personalized cardiac computational models: From clinical data to simulation of infarct-related ventricular tachycardia. Front. Physiol..

[29] Bayer JD, Blake RC, Plank G, Trayanova NA (2012). A novel rule-based algorithm for assigning myocardial fiber orientation to computational heart models. Ann. Biomed. Eng..

[30] Durrer D (1970). Total excitation of the isolated human heart. Circulation.

[31] Barber F, García-Fernández I, Lozano M, Sebastian R (2018). Automatic estimation of Purkinje-myocardial junction hot-spots from noisy endocardial samples: A simulation study. Int. J. Numer. Methods Biomed. Eng..

[32] Gepstein L, Hayam G, Ben-Haim SA (1997). A novel method for nonfluoroscopic catheter-based electroanatomical mapping of the heart: In vitro and in vivo accuracy results. Circulation.

[33] Sundnes J (2006). On the computational complexity of the bidomain and the monodomain models of electrophysiology. Ann. Biomed. Eng..

[34] Tomek J (2019). Development, calibration, and validation of a novel human ventricular myocyte model in health, disease, and drug block. Elife.

[35] Tomek J, Bueno-Orovio A, Rodriguez B (2020). ToR-ORd-dynCl: An update of the ToR-ORd model of human ventricular cardiomyocyte with dynamic intracellular chloride. BioRxiv.

[36] Sachetto Oliveira R (2018). Performance evaluation of GPU parallelization, space-time adaptive algorithms, and their combination for simulating cardiac electrophysiology. Int. J. Numer. Methods Biomed. Eng..

[37] ten Tusscher KH, Noble D, Noble P-J, Panfilov AV (2004). A model for human ventricular tissue. Am. J. Physiol.-Heart Circul. Physiol..

[38] O’Hara T, Virág L, Varró A, Rudy Y (2011). Simulation of the undiseased human cardiac ventricular action potential: Model formulation and experimental validation. PLoS Comput. Biol..

[39] Stewart P (2009). Mathematical models of the electrical action potential of Purkinje fibre cells. Philos. Trans. R. Soc. A: Math. Phys. Eng. Sci..

[40] Schoenberg M, Dominguez G, Fozzard HA (1975). Effect of diameter on membrane capacity and conductance of sheep cardiac Purkinje fibers. J. Gen. Physiol..

[41] Banerjee A (2021). A completely automated pipeline for 3d reconstruction of human heart from 2d cine magnetic resonance slices. Phil. Trans. R. Soc. A.

[42] Kucera JP, Kléber AG, Rohr S (1998). Slow conduction in cardiac tissue, II. Circ. Res..

[43] Kucera JP, Rudy Y (2001). Mechanistic insights into very slow conduction in branching cardiac tissue. Circ. Res..

[44] Behradfar E, Nygren A, Vigmond EJ (2014). The role of Purkinje-myocardial coupling during ventricular arrhythmia: A modeling study. PLoS ONE.

[45] Morley GE (2005). Reduced intercellular coupling leads to paradoxical propagation across the Purkinje-ventricular junction and aberrant myocardial activation. Proc. Natl. Acad. Sci..

[46] Berenfeld O, Jalife J (1998). Purkinje-muscle reentry as a mechanism of polymorphic ventricular arrhythmias in a 3-dimensional model of the ventricles. Circ. Res..

[47] Walz, T. P., Azzolin, L., Chleilat, E., Berg, L. & Arevalo, H. Potential roles of purkinje fibers in ischemia-induced arrhythmias. In *2020 Computing in Cardiology* 1–4 (IEEE, 2020).

[48] Haissaguerre M (2022). Purkinje network and myocardial substrate at the onset of human ventricular fibrillation: Implications for catheter ablation. Eur. Heart J..

[49] Tabereaux PB (2007). Activation patterns of Purkinje fibers during long-duration ventricular fibrillation in an isolated canine heart model. Circulation.

[50] Cherry EM, Fenton FH (2012). Contribution of the Purkinje network to wave propagation in the canine ventricle: Insights from a combined electrophysiological-anatomical model. Nonlinear Dyn..

