# Supplementary material for: Enhanced optimization-based method for the generation of patient-specific models of Purkinje networks
Source: Sci Rep. 2023 Jul 21;13:11788. doi: 10.1038/s41598-023-38653-1 (PMC10362015; doi:10.1038/s41598-023-38653-1)
Supplement: Supplementary file 1 — Supplementary Information. [file 41598_2023_38653_MOESM1_ESM.pdf]

Enhanced Optimization-Based Method for the Generation of Patient-Specific Models of Purkinje Networks

Lucas Arantes Berg, Bernardo Martins Rocha, Rafael Sachetto Oliveira, Rafael Sebastian, Blanca Rodriguez, Rafael Alves Bonfim de Queiroz, Elizabeth M. Cherry, Rodrigo Weber dos Santos

A Supplementary Material

A.1 Geometrical concepts and data structures

To properly evaluate the geometrical features of our PN models, the mean and standard deviation of the branch size and angle of the bifurcations are computed together with the total number of branches and bifurcations. The concept of a segment in the tree is equivalent to an edge in their corresponding graph representing the PN, while a branch is a set of segments between bifurcations. Within this context, the PN is represented with a graph data structure, allowing each segment node to access its parent and left/right off-springs. In Figure S1, the main geometrical concepts of the method are illustrated together with the data structure utilized to store the nodes and segments of the PN.

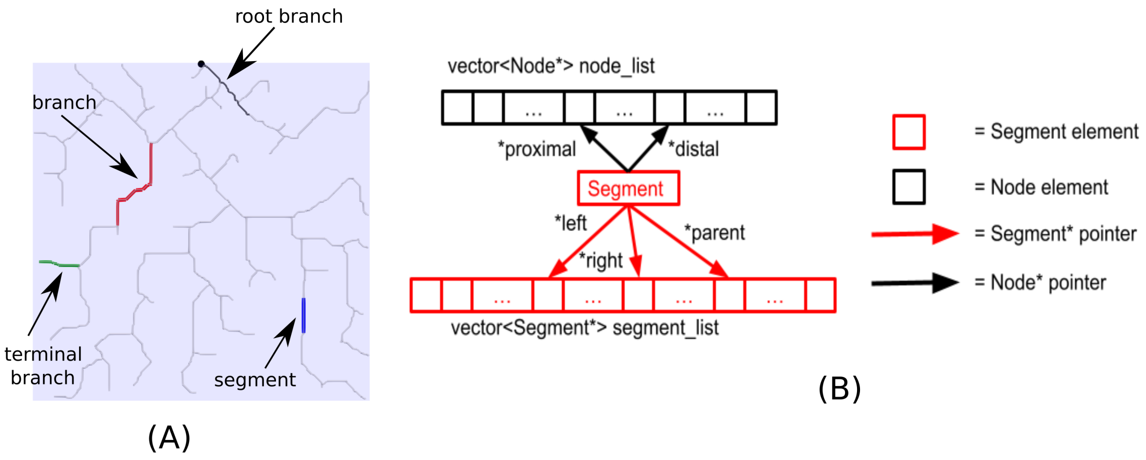

Figure S1 . Illustration showing the different geometrical concepts related to the method alongside the data structure used to store the nodes and segments of the PN. In panel (A), the concept of a segment and branch is highlighted in blue and red, respectively. In addition, the initial root branch of the PN is colored in black, while an example of a terminal branch is presented in green. In panel (B), the data structures used to store a PN are illustrated. There are two arrays, one of nodes and another of segments which keep track of the current state of the PN. Each segment has access to its parent and left/right off-springs, which are all pointers to the segment structure. Moreover, each segment has two pointers to the two nodes that define the segment. Using these pointers is possible to retrieve the proximal and distal coordinates of any segment.

A.2 Simplified mesh generation

A.2.1 Biventricular surface

The simplified biventricular mesh was constructed by taking the differences between three ellipsoids, one for the Left ventricle (LV) and Right ventricle (RV) endocardium and one for the epicardium. The following equation gives an ellipsoid that is not centered on the domain origin:

$$\frac{(x-c_x)^2}{a^2}+\frac{(y-c_y)^2}{b^2}+\frac{(z-c_z)^2}{c^2}=1, \tag{1}$$

where c_x, c_y and c_z are the center coordinates.

The LV surface is built by taking the difference between two ellipsoids. The LV epicardium given by (3) is subtracted from the LV endocardium given by (2). Similarly, the RV surface is built by taking the difference between three ellipsoids. The RV epicardium given by (5) is subtracted from the RV endocardium (4) and the LV surface previously calculated. Next, the biventricular surface is assembled by joining the LV and RV surfaces and cutting the base plane defined by $x = 0$. Finally, the

mesh is translated to the origin and scaled to micrometers.

$$\frac{x^2}{1.5^2}+\frac{y^2}{0.5^2}+\frac{z^2}{0.5^2}=1, \tag{2}$$

$$\frac{x^2}{2^2}+\frac{y^2}{1^2}+\frac{z^2}{1^2}=1, \tag{3}$$

$$\frac{x^2}{1.45^2}+\frac{(y-0.5)^2}{1.25^2}+\frac{z^2}{0.75^2}=1, \tag{4}$$

$$\frac{x^2}{1.75^2}+\frac{(y-0.5)^2}{1.5^2}+\frac{z^2}{1^2}=1. \tag{5}$$

A.2.2 Purkinje network

To build the reference Purkinje network (PN) for the simplified mesh, we use the fractal algorithm from[1] with the parameters given by Table S1. Regarding the parameter description from the fractal algorithm by *Costabal et al.*[1], the Root and Second node coordinates are utilized to give the initial growth direction of the first segment. Fascicle angle and length are related to the generation of the first segment by providing information if there will be a bifurcation after the first segment using a specified angle. If the Fascicle angle is zero, a linear fascicle segment grows with a designated length from the distal position of the first segment. The Repulsive parameter regulates the branch curvature: the larger the repulsion parameter, the more the branches repel each other. The branch angle controls the mean branch bifurcation angles. The number of iterations configures the number of growth iterations. After the networks were generated, the structures were translated to the origin and scaled to micrometers.

Parameter name	LV	RV
Length of the first branch (mm)	10	15
Mean length of the branches (mm)	4	4
Number of iterations	5	5
Branch angle (degrees)	26	26
Repulsive parameter	0.1	0.1
Root coordinates (mm)	[2.40, 37.15, 26.35]	[2.40, 49.19, 20.02]
Second node coordinates (mm)	[4.84, 36.95, 26.43]	[11.20, 48.81, 21.99]
Fascicles angle (degrees)	74.48	0
Fascicles length (mm)	10	10

Table S1 . Parameters used for the generation of the reference PN of the Simplified mesh.

A.3 Purkinje-Ventricular-Junction delay tuning

To model the Purkinje-Ventricular-Junctions (PVJs) sites, an additional current, I_{PVJ}^{tiss}, was included on the tissue cells that are coupled to the terminal cells from the PN as described in the following equation:

$$I_{PVJ}^{tiss}=\sum_{i=0}^{N_{PVJ}}\frac{(V^{purk}-V_i^{tiss})}{R_{PVJ}}, \tag{6}$$

where V^{purk} is the transmembrane potential of the terminal Purkinje cells, V_i^{tiss} is the transmembrane potential of the ventricular cells i attached to the Purkinje cell, R_{PVJ} is a fixed-resistance and N_{PVJ} is the maximum number of ventricular cells that are inside the PVJ site. This additional current is included on the right hand side of the associate linear system of the ventricular domain only for the ventricular cells related to the coupling.

Similarly, for the Purkinje cells a current, I_{PVJ}^{purk}, coming from the ventricular cells that are coupled to the terminal Purkinje cell, is included on the right hand side of the associate linear system of the Purkinje domain only for the terminal Purkinje cell related to the coupling and is given by the following equation:

$$I_{PVJ}^{purk}=\sum_{i=0}^{N_{PVJ}}\frac{(V_i^{tiss}-V^{purk})}{R_{PVJ}}, \tag{7}$$

In Figure S2, we illustrate how the Purkinje coupling was implemented and in Figure S3 how the experiment was done.

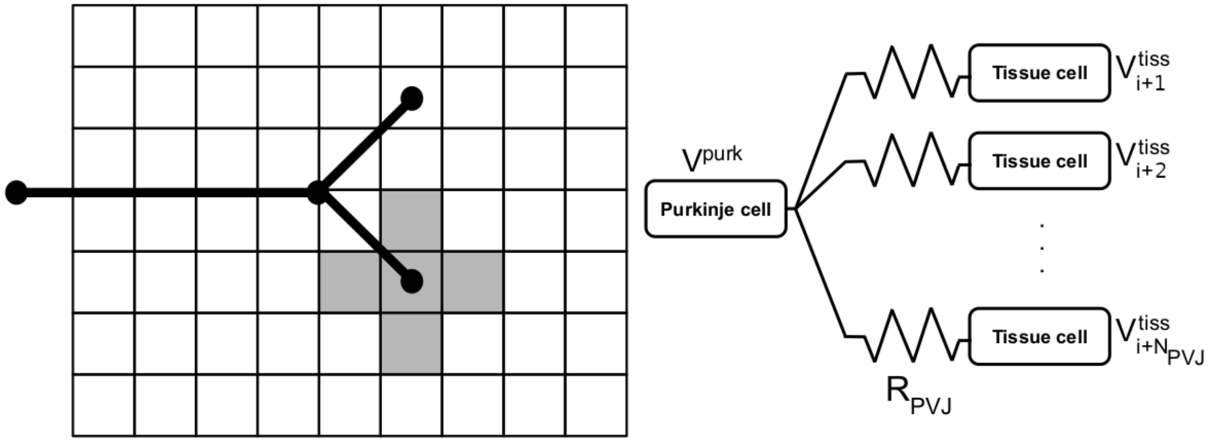

Figure S2 . Illustration of the Purkinje coupling model. The terminal Purkinje cells are linked to the nearest N_{PVJ} ventricular cells by a fixed resistance R_{PVJ} and two additional currents I_{PVJ}^{purk} and I_{PVJ}^{tiss} are computed following equations (6) and (7) for only the Purkinje and ventricular cells that are related to the coupling.

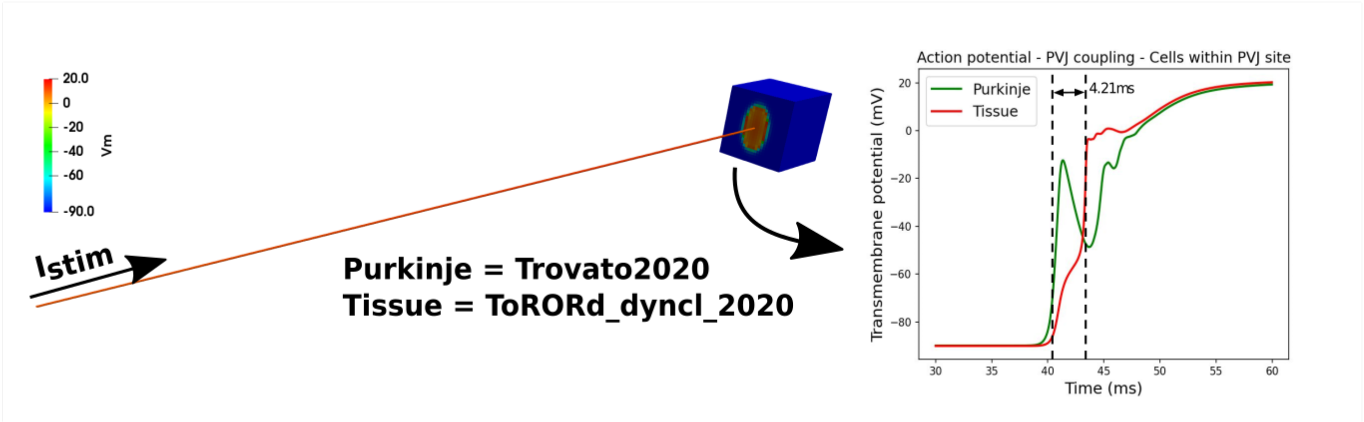

Figure S3 . Experiment to calibrate the Purkinje-Ventricular-Junction delay in the anterograde direction. A single $10cm$ Purkinje fiber stimulates a face of a tissue block ($1cm^3$). The PVJ delay in the anterograde direction is measured by the difference between the LAT of the ventricular cells and Purkinje cell related to the coupling. The Purkinje cells are modeled using the Trovato human Purkinje model[2] and the ventricular cells using the recent version of the Tomek human ventricular model[3]. The action potential traces from both the Purkinje and one of the ventricular cells of the coupling are depicted alongside the PVJ delay when $R_{PVJ} = 900k\Omega$ and $N_{PVJ} = 60$ in the right panel.

Applying different combinations of R_{PVJ}, and N_{PVJ} we measured the PVJ delay as Table S2 illustrates.

(R_{PVJ}, N_{PVJ})	**40**	**45**	**50**	**55**	**60**
**300**	2.43	2.15	1.7	1.69	1.35
**400**	5.39	2.97	2.31	1.86	1.75
**500**	block	block	2.84	2.38	2.17
**600**	block	block	3.91	2.85	2.51
**700**	block	block	block	3.6	**3.1**
**800**	block	block	block	4.61	3.5
**900**	block	block	block	block	4.21
$\{k\Omega\}$					

Table S2 . Results of the PVJ calibration experiments show how the anterograde PVJ delay varies in milliseconds for different values of R_{PVJ} and N_{PVJ}. When the Purkinje fiber could not stimulate the ventricular cells, we define the PVJ delay to ∞ and the tag block in the table.

Based on the results from Table S2, the Purkinje coupling parameters were set to $R_{PVJ} = 700k\Omega$ and $N_{PVJ} = 60$, generating a PVJ delay of approximately 3 ms in the anterograde direction, which is within the acceptable range[4].

A.4 Geometric results

In the following section the geometrical results from the 10 best PNs regarding the three biventricular meshes are presented. To evaluate the PNs geometrically the branch size (millimeters), bifurcation angle (^{o}) and the total number of branches and angles in the trees are calculated considering the mean and standard deviation of its values.

A.4.1 Simplified mesh

In Table S3 the geometrical results for the Simplified mesh are illustrated. As can be seen in this table, the values in terms of branch size demonstrate that the RV trees have a longer size when compared to the LV ones. When the average and standard deviation values are compared to the reference PN, we can noticed that the values of branch size for the 10 best networks can vary depending on the endocardium region where trees were generated. For the bifurcation angle measurement in this mesh, we could verify that the average values from both biventricular regions were close to the reference values and that the angles for the RV trees have more acute shape. Finally, for the total number of branches and bifurcations in the trees the values were exactly the same as the reference ones, which was already expected since all the generated PNs have the same number of terminals as the reference. This can also be verified by the fact that all the active PVJs are connected to the minimum network.

LV	Branch size (mm)	Angle(^{o})	#Branches	#Angles
**Reference**	**2.93 ± 1.71**	**52.02 ± 3.95**	**85**	**42**
**PK1**	4.16 ± 4.04	42.54 ± 28.67	85	42
**PK2**	4.71 ± 4.64	46.33 ± 32.45	85	42
**PK3**	4.00 ± 4.05	50.71 ± 32.72	85	42
**PK4**	4.49 ± 4.30	53.57 ± 31.39	85	42
**PK5**	4.15 ± 3.59	49.64 ± 34.94	85	42
**PK6**	4.38 ± 4.22	50.23 ± 34.02	85	42
**PK7**	4.80 ± 4.73	52.51 ± 30.30	85	42
**PK8**	4.47 ± 3.92	41.02 ± 32.46	85	42
**PK9**	4.57 ± 4.10	49.02 ± 32.55	85	42
**PK10**	4.27 ± 4.29	59.57 ± 41.34	85	42
RV	Branch size (mm)	Angle(^{o})	#Branches	#Angles
**Reference**	**3.41 ± 3.00**	**34.23 ± 0.28**	**39**	**19**
**PK1**	6.16 ± 6.27	35.32 ± 26.07	39	19
**PK2**	6.64 ± 7.34	40.41 ± 27.69	39	19
**PK3**	6.16 ± 6.26	35.29 ± 26.09	39	19
**PK4**	6.67 ± 6.51	39.04 ± 26.70	39	19
**PK5**	6.70 ± 7.20	33.86 ± 28.10	39	19
**PK6**	6.12 ± 5.96	45.90 ± 27.21	39	19
**PK7**	6.57 ± 7.28	36.47 ± 30.09	39	19
**PK8**	7.51 ± 8.61	35.54 ± 25.65	39	19
**PK9**	7.06 ± 7.92	34.47 ± 29.19	39	19
**PK10**	7.05 ± 7.90	35.82 ± 28.26	39	19

Table S3 . Results for the geometric features from the best 10 Purkinje networks of the Simplified mesh. For this particular mesh, the Reference Purkinje network was generated using the fractal method by *Costabal et al.*[1].

A.4.2 Canine mesh

Analyzing the geometrical results for the Canine mesh in Table S4 we noticed that the average values for the branch size were closer to the reference ones in both biventricular regions when compared to the previous results from the Simplified mesh. In terms of bifurcation angle, the values from the generated PNs presented a high variability indicating that the complexity of the endocardium surface can affect the shape of the angles in the trees. Similarly, to the Simplified mesh results the values for the total number of branches and bifurcations angles match exactly the reference values since the minimum networks generated by the method have the same number of terminals as the gold standard trees.

LV	Branch size (mm)	Angle(^{o})	#Branches	#Angles
**Reference**	**3.56 ± 3.09**	**89.48 ± 44.47**	**259**	**129**
**PK1**	4.07 ± 4.01	54.30 ± 39.14	259	129
**PK2**	4.24 ± 3.79	50.57 ± 39.29	259	129
**PK3**	4.11 ± 4.24	61.16 ± 45.47	259	129
**PK4**	4.19 ± 4.18	50.65 ± 38.88	259	129
**PK5**	4.22 ± 4.25	56.21 ± 36.68	259	129
**PK6**	4.31 ± 4.43	53.31 ± 41.84	259	129
**PK7**	3.97 ± 3.83	58.95 ± 39.94	259	129
**PK8**	4.26 ± 3.91	56.69 ± 39.25	259	129
**PK9**	4.18 ± 3.79	57.49 ± 41.04	259	129
**PK10**	4.28 ± 4.17	56.23 ± 37.51	259	129
RV	Branch size (mm)	Angle(^{o})	#Branches	#Angles
**Reference**	**3.43 ± 3.61**	**76.58 ± 39.57**	**195**	**97**
**PK1**	3.77 ± 4.20	60.00 ± 43.87	195	97
**PK2**	4.06 ± 4.75	55.61 ± 44.73	195	97
**PK3**	3.75 ± 4.74	51.21 ± 38.99	195	97
**PK4**	4.08 ± 4.86	48.54 ± 40.01	195	97
**PK5**	4.38 ± 6.14	53.63 ± 42.20	195	97
**PK6**	4.31 ± 4.64	58.97 ± 47.72	195	97
**PK7**	4.21 ± 5.49	45.15 ± 32.27	195	97
**PK8**	4.12 ± 4.81	51.21 ± 43.23	195	97
**PK9**	3.90 ± 4.39	53.86 ± 41.50	195	97
**PK10**	4.08 ± 4.82	49.08 ± 39.90	195	97

Table S4 . Results for the geometric features from the best 10 Purkinje networks of the Canine mesh. In the case of the Canine mesh, the Reference network was provided from the works[5,6].

A.4.3 Patient-specific mesh

In Table S5 the results for the patient-specific could not be compared to a reference PN since only the location and LAT of the active PVJs sites were available. However, important topological observations can be made from the generated PNs. In terms of branch size, the LV and RV trees for this particular mesh present the highest values. This can be justified due to the fact that the Patient-specific mesh have the largest endocardium volume when it is compared to the Simplified and Canine meshes leading to prolonged branches in either biventricular regions.

LV	Branch size (mm)	Angle(°)	#Branches	#Angles
**PK1**	16.10 ± 17.05	54.35 ± 32.09	63	31
**PK2**	18.55 ± 19.90	45.82 ± 30.01	63	31
**PK3**	17.20 ± 13.53	51.62 ± 32.52	63	31
**PK4**	15.90 ± 15.54	59.75 ± 33.06	63	31
**PK5**	17.08 ± 19.74	58.29 ± 40.93	63	31
**PK6**	15.20 ± 17.38	59.11 ± 40.69	63	31
**PK7**	15.65 ± 16.87	53.76 ± 33.22	63	31
**PK8**	16.04 ± 16.50	60.20 ± 41.07	63	31
**PK9**	16.15 ± 16.38	49.70 ± 32.23	63	31
**PK10**	16.96 ± 19.91	59.88 ± 35.55	63	31
RV	Branch size (mm)	Angle(°)	#Branches	#Angles
**PK1**	18.53 ± 15.86	65.55 ± 48.63	33	16
**PK2**	17.92 ± 15.23	65.50 ± 47.24	33	16
**PK3**	19.57 ± 16.93	64.89 ± 43.16	33	16
**PK4**	18.30 ± 16.52	55.73 ± 45.12	33	16
**PK5**	19.25 ± 17.46	45.75 ± 33.48	33	16
**PK6**	17.38 ± 12.80	64.60 ± 48.96	33	16
**PK7**	19.85 ± 15.40	62.86 ± 42.40	33	16
**PK8**	19.65 ± 13.65	58.54 ± 41.07	33	16
**PK9**	17.52 ± 14.42	63.71 ± 43.60	33	16
**PK10**	19.00 ± 18.00	59.51 ± 45.24	33	16

Table S5 . Results for the geometric features from the best 10 Purkinje networks of the Patient-specific mesh.

A.5 Activation results

In the next section the activation results from the 10 best PNs of the three biventricular meshes are depicted. To evaluate the PNs electrically the minimum and maximum LAT (milliseconds), maximum LAT error (milliseconds), RMSE (milliseconds), RRMSE (%), $\varepsilon < 2ms$ (%) and $\varepsilon < 5ms$ (%) are evaluated at the active PVJ sites in the trees.

A.5.1 Simplified mesh

In Table S6 the activation results for the Simplified mesh are illustrated. As can be seen in the table, the generated PNs presented a minimum and maximum LAT close to the reference values in both biventricular regions. When the maximum LAT error is analyzed the trees presented a good accuracy with errors below $3ms$. This observation is also sustained by RMSE error results below $1ms$ and almost the majority of the active PVJs being connected within a LAT error tolerance below $2ms$.

LV	minLAT (ms)	maxLAT (ms)	maxError (ms)	RMSE (ms)	RRMSE (%)	$\varepsilon < 2ms$ (%)	$\varepsilon < 5ms$ (%)
**Reference**	**11.83**	**18.25**	**-**	**-**	**-**	**-**	**-**
PK1	11.84	19.04	1.01	0.28	1.84	100.00	100.00
PK2	12.14	19.05	0.80	0.33	2.16	100.00	100.00
PK3	11.82	19.45	1.20	0.37	2.43	100.00	100.00
PK4	11.82	19.39	1.13	0.37	2.40	100.00	100.00
PK5	11.85	19.03	1.54	0.40	2.59	100.00	100.00
PK6	11.78	19.30	1.72	0.42	2.76	100.00	100.00
PK7	11.85	19.32	1.87	0.45	2.92	100.00	100.00
PK8	11.82	19.06	2.55	0.46	3.01	97.67	100.00
PK9	11.79	19.07	2.69	0.47	3.07	97.67	100.00
PK10	11.85	19.19	2.47	0.48	3.13	97.67	100.00
**RV**	**minLAT (ms)**	**maxLAT (ms)**	**maxError (ms)**	**RMSE (ms)**	**RRMSE (%)**	$\varepsilon < 2ms$ (%)	$\varepsilon < 5ms$ (%)
**Reference**	**14.68**	**17.50**	**-**	**-**	**-**	**-**	**-**
PK1	14.27	17.75	1.08	0.37	2.28	100.00	100.00
PK2	14.19	17.73	1.10	0.37	2.27	100.00	100.00
PK3	14.27	17.75	1.08	0.37	2.28	100.00	100.00
PK4	14.21	17.77	1.10	0.38	2.34	100.00	100.00
PK5	14.23	17.79	1.10	0.38	2.33	100.00	100.00
PK6	14.25	17.75	1.11	0.38	2.36	100.00	100.00
PK7	14.20	17.80	1.11	0.39	2.42	100.00	100.00
PK8	14.28	17.80	1.18	0.41	2.52	100.00	100.00
PK9	14.28	17.81	1.18	0.41	2.54	100.00	100.00
PK10	14.28	17.80	1.18	0.41	2.53	100.00	100.00

Table S6 . Results for the activation features from the best 10 Purkinje networks of the Simplified mesh. For this particular mesh, the Reference Purkinje network was generated using the fractal method by *Costabal et al.*[1]

A.5.2 Canine mesh

In Table S7 the activation results for the Canine mesh are highlighted. Based on these results, for the minimum and maximum LAT, the generated PNs demonstrate a good accuracy with values close to the reference ones in both biventricular regions. In terms of maximum LAT error, we noticed that the LV trees have the highest values when compared to the RV trees which can be justified due to the fact that are more active PVJs in the LV region of this mesh than the RV. Analyzing the RMSE, RRMSE, $\varepsilon < 2ms$ and $\varepsilon < 5ms$ metrics the PNs from both regions presented good accuracy with errors equivalent to the results from the Simplified mesh.

LV	minLAT (ms)	maxLAT (ms)	maxError (ms)	RMSE (ms)	RRMSE (%)	$\varepsilon < 2ms$ (%)	$\varepsilon < 5ms$ (%)
**Reference**	**9.86**	**40.16**	-	-	-	-	-
PK1	10.10	38.96	4.22	0.64	2.49	97.69	100.00
PK2	9.90	40.35	3.74	0.65	2.54	98.46	100.00
PK3	7.94	40.33	4.31	0.68	2.67	97.69	100.00
PK4	9.93	38.70	4.39	0.74	2.90	96.92	100.00
PK5	9.49	40.09	4.80	0.74	2.90	98.46	100.00
PK6	9.68	40.19	4.37	0.74	2.91	96.92	100.00
PK7	9.44	40.29	4.71	0.74	2.90	97.69	100.00
PK8	9.63	40.38	5.07	0.76	2.99	97.69	99.23
PK9	9.85	40.44	4.98	0.77	3.00	98.46	100.00
PK10	9.72	40.16	4.38	0.80	3.13	96.92	100.00
**RV**	**minLAT (ms)**	**maxLAT (ms)**	**maxError (ms)**	**RMSE (ms)**	**RRMSE (%)**	$\varepsilon < 2ms$ (%)	$\varepsilon < 5ms$ (%)
**Reference**	**9.76**	**53.93**	-	-	-	-	-
PK1	10.44	53.88	1.59	0.51	1.41	100.00	100.00
PK2	10.04	53.92	2.31	0.54	1.48	98.98	100.00
PK3	10.08	53.84	1.94	0.59	1.63	100.00	100.00
PK4	9.79	53.92	2.10	0.60	1.64	98.98	100.00
PK5	9.81	54.11	2.26	0.63	1.71	95.92	100.00
PK6	10.09	53.93	1.93	0.64	1.75	100.00	100.00
PK7	9.79	53.89	2.01	0.64	1.76	97.96	100.00
PK8	9.79	53.92	1.94	0.67	1.84	100.00	100.00
PK9	10.54	53.89	2.27	0.67	1.83	97.96	100.00
PK10	12.06	53.99	2.33	0.68	1.85	96.94	100.00

Table S7 . Results for the activation features from the best 10 Purkinje networks of the Canine mesh. In the case of the Canine mesh, the Reference network was provided from the works[5,6].

A.5.3 Patient-specific mesh

In Table S8 the activation results for the Patient-specific mesh are presented. Similarly to the previous meshes, the generated PNs performed well in terms of minimum and maximum LAT leading to values close to the reference values. Only in the RV region of the Patient-specific mesh we found that a particular PVJ could not be connected with a LAT error below $5ms$. When the other electrical measurements are analyzed, we noticed that the LV trees presented a better approximation to the reference LAT at the active PVJ sites. In addition, an interesting observation that can be made for the RV trees is that the 10 best networks for this region presented a very similar LAT even with a very distinctly topology.

LV	minLAT (ms)	maxLAT (ms)	maxError (ms)	RMSE (ms)	RRMSE (%)	$\varepsilon < 2ms$ (%)	$\varepsilon < 5ms$ (%)
**Reference**	**36.13**	**123.77**	-	-	-	-	-
PK1	35.55	123.75	3.63	1.16	1.50	90.62	100.00
PK2	35.96	123.76	3.70	1.22	1.59	87.50	100.00
PK3	35.96	123.70	4.02	1.32	1.72	90.62	100.00
PK4	35.55	123.70	3.46	1.36	1.76	87.50	100.00
PK5	35.62	125.55	5.47	1.52	1.97	84.38	96.88
PK6	36.40	123.93	3.53	1.54	2.00	78.12	100.00
PK7	35.70	123.73	4.12	1.54	2.00	84.38	100.00
PK8	35.70	123.05	4.21	1.77	2.29	78.12	100.00
PK9	36.40	124.01	4.94	1.79	2.32	81.25	100.00
PK10	35.55	125.49	7.40	1.82	2.36	84.38	96.88
**RV**	**minLAT (ms)**	**maxLAT (ms)**	**maxError (ms)**	**RMSE (ms)**	**RRMSE (%)**	$\varepsilon < 2ms$ (%)	$\varepsilon < 5ms$ (%)
**Reference**	**35.71**	**60.87**	-	-	-	-	-
PK1	31.05	60.88	6.83	1.79	3.90	94.12	94.12
PK2	31.05	60.78	6.85	1.81	3.95	94.12	94.12
PK3	31.05	61.34	6.84	1.81	3.93	94.12	94.12
PK4	31.05	60.82	6.85	1.81	3.93	94.12	94.12
PK5	31.05	60.77	6.86	1.82	3.96	94.12	94.12
PK6	31.05	60.57	6.83	1.82	3.96	94.12	94.12
PK7	31.05	60.80	6.85	1.82	3.97	94.12	94.12
PK8	31.05	60.92	6.83	1.82	3.97	94.12	94.12
PK9	31.05	60.69	6.83	1.83	3.99	94.12	94.12
PK10	31.05	61.59	6.85	1.83	4.00	94.12	94.12

Table S8 . Results for the activation features from the best 10 Purkinje networks of the Patient-specific mesh. The LAT values of the Patient-specific mesh were estimated by the CARTO ^{TM} maps.

A.6 Monodomain results

A.6.1 Simplified mesh

In Figure S4 the results of the monodomain simulations for the Simplified mesh are depicted. Firstly, in Figure S4A, it is illustrated the baseline LAT calculated when the reference PN is coupled to the biventricular tissue. Secondly, in Figures S4B and S4C, it is shown the LAT obtained when the best and worst PN generated by the method are coupled to the biventricular tissue, respectively. Finally, in Figure S4D and S4E, it is presented the absolute difference between the baseline and approximate LAT given by the PNs, where the results for best PN is on panel D and the worst PN on panel E.

From the results of Figure S4, we noticed that both the best and worst PNs of our comparison set show a feasible LAT map for the Simplified mesh. As can be seen in panels D and E of Figure S4, the best and worst PNs present a maximum absolute LAT difference around $3ms$, which is acceptable in terms of LAT matching.

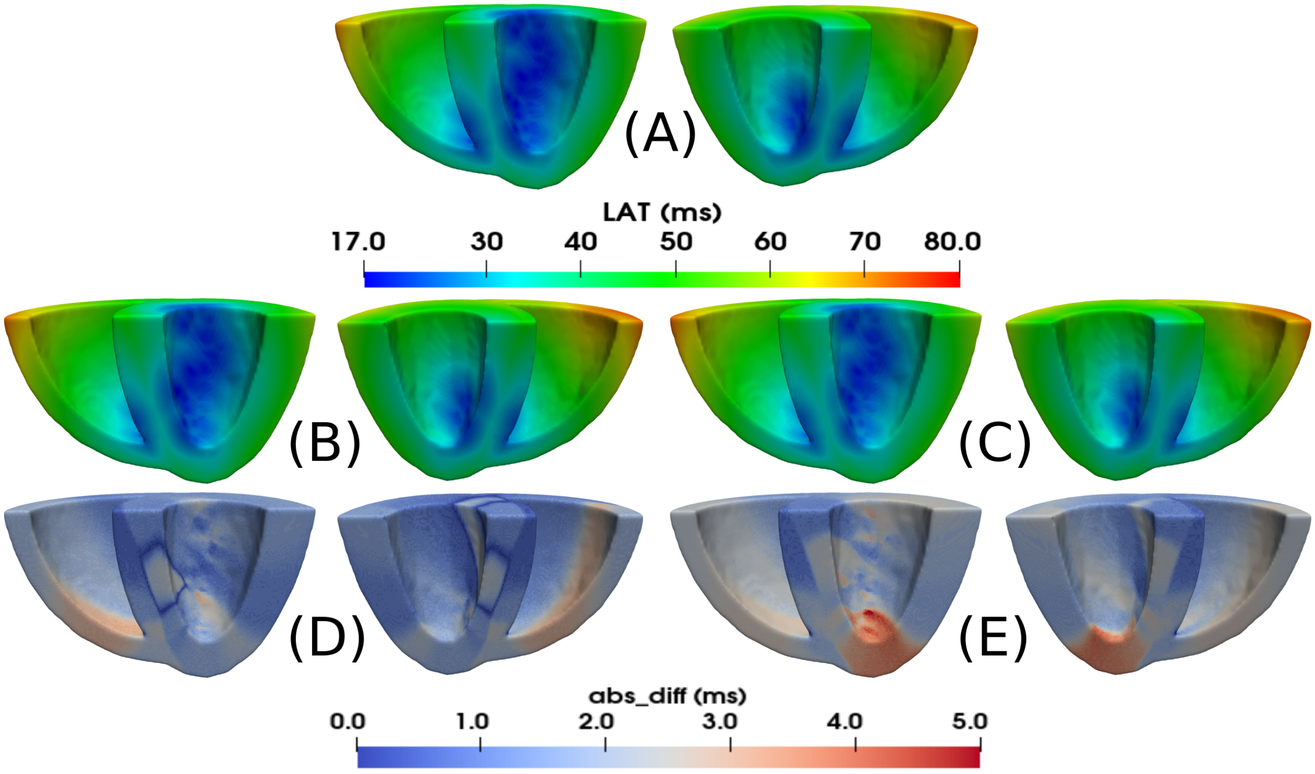

Figure S4 . Results for the LAT maps when a coupled monodomain simulation is executed over the Simplified mesh. First, in the top panel (A), the active PVJs are activated following the LAT provided by the reference PN. Next, in the middle panels (B) and (C), the LAT maps of the best and worst PNs generated by our method are depicted, respectively. Finally, in the bottom panels (D) and (E), the absolute difference between the LAT maps from the best and worst PNs is calculated, respectively.

A.6.2 Canine mesh

In Figure S5 the results of the monodomain simulations for the Canine mesh are illustrated. Initially, in Figure S5A, it is depicted the baseline LAT calculated when the reference PN is coupled to the biventricular tissue. Next, in Figures S5B and S5C, it is shown the LAT obtained when the best and worst PNs generated by our method are coupled to the biventricular tissue, respectively. Lastly, in Figures S5D and S5E, it is presented the absolute difference between the baseline and approximate LAT given by the PNs, where the results for best PN is on panel D and the worst PN on panel E.

From the LAT and absolute difference maps in Figure S5, we can conclude that the best PN was able to activate the ventricular tissue similar to the reference LAT in almost all the biventricular tissue, except in a specific region of the RV. On the other hand, the worst PN could not activate several PVJs at the correct LAT even with a good approximation when the cable equation was used. This behaviour could be related to the PVJ coupling parameters used in the monodomain simulation. Mainly, from these results, we can conclude that certain PVJs must activated with a LAT very close to each other to stimulate these regions. In addition, as can be seen in these results, the PVJ delay plays an important role in ventricular activation and depending on certain conditions might be responsible for different LAT.

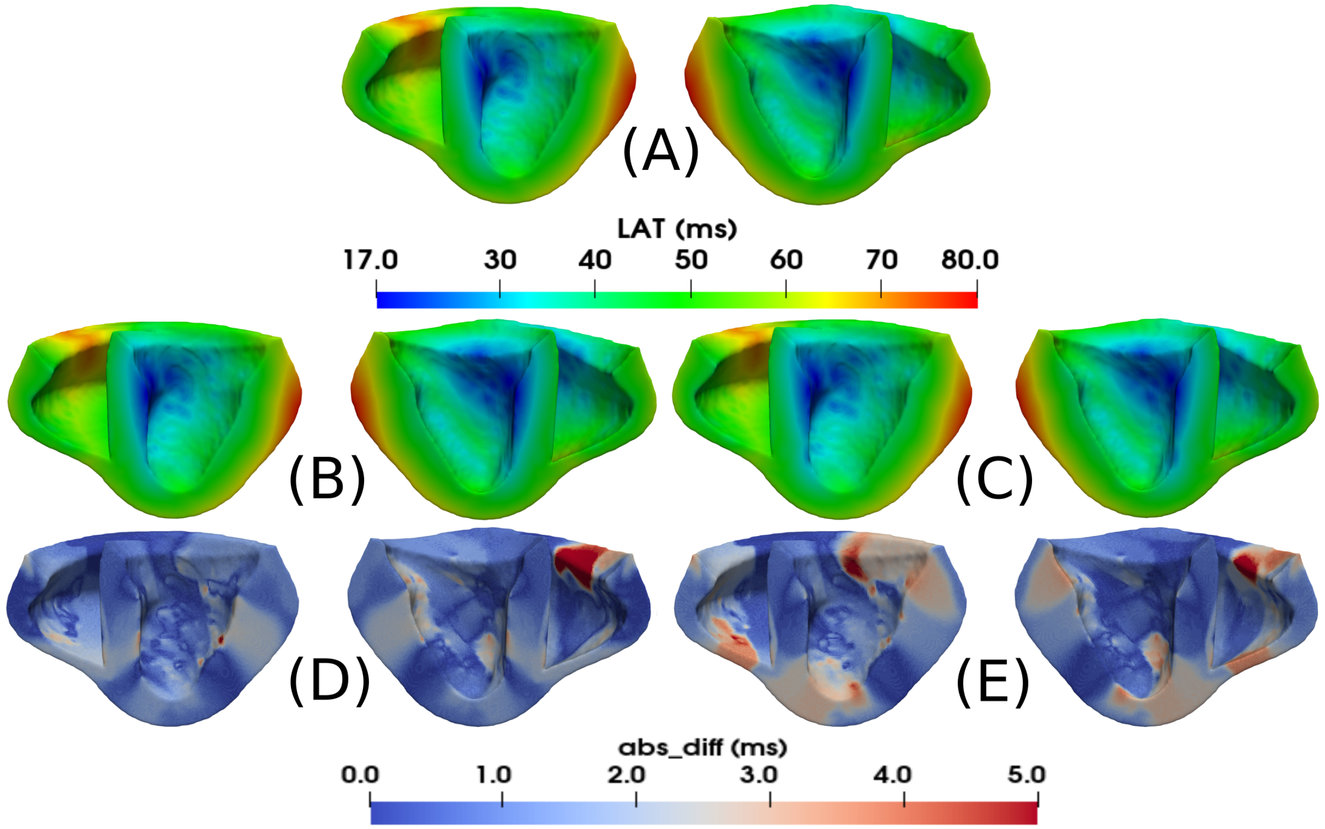

Figure S5 . Results for the LAT maps when a coupled monodomain simulation is executed over the Canine mesh. First, in the top panel (A), the active PVJs are activated following the LAT provided by the reference PN. Next, in the middle panels (B) and (C), the LAT maps of the best and worst PNs generated by our method are depicted, respectively. Finally, in the bottom panels (D) and (E), the absolute difference between the LAT maps from the best and worst PNs is calculated, respectively.

A.6.3 Patient-specific mesh - Isolating the effects of the PNs

To further investigate the differences between the two simulated LAT maps generated by the best and worst PNs in the patient-specific mesh, which is depicted in Figure 6 from the main manuscript, we consider in Figure S6 the difference between the two respective LAT maps to verify if the cause of the errors are associated to the ventricular propagation model since the effects of the PN will be isolated.

As can be seen in Figure S6 we show the LAT maps generated when using the best and worst Purkinje networks for the patient-specific mesh together with the absolute LAT difference calculated between the two maps. As can be see in this figure and also in Figure 6 from the main manuscript, some effects of the Purkinje are isolated, like the region around a PVJ that could not be activated properly in the RV by both networks. However, from Figure S6 we can noticed that the activation of the LV is different between the two networks around the apex region with an absolute difference around $3ms$ and $4ms$, which confirms that the errors are not only caused by the ventricular propagation model but also by the actual difference in the LAT at the PVJ sites of the two PNs which is sustain by the fact that the topology of the two PNs are different.

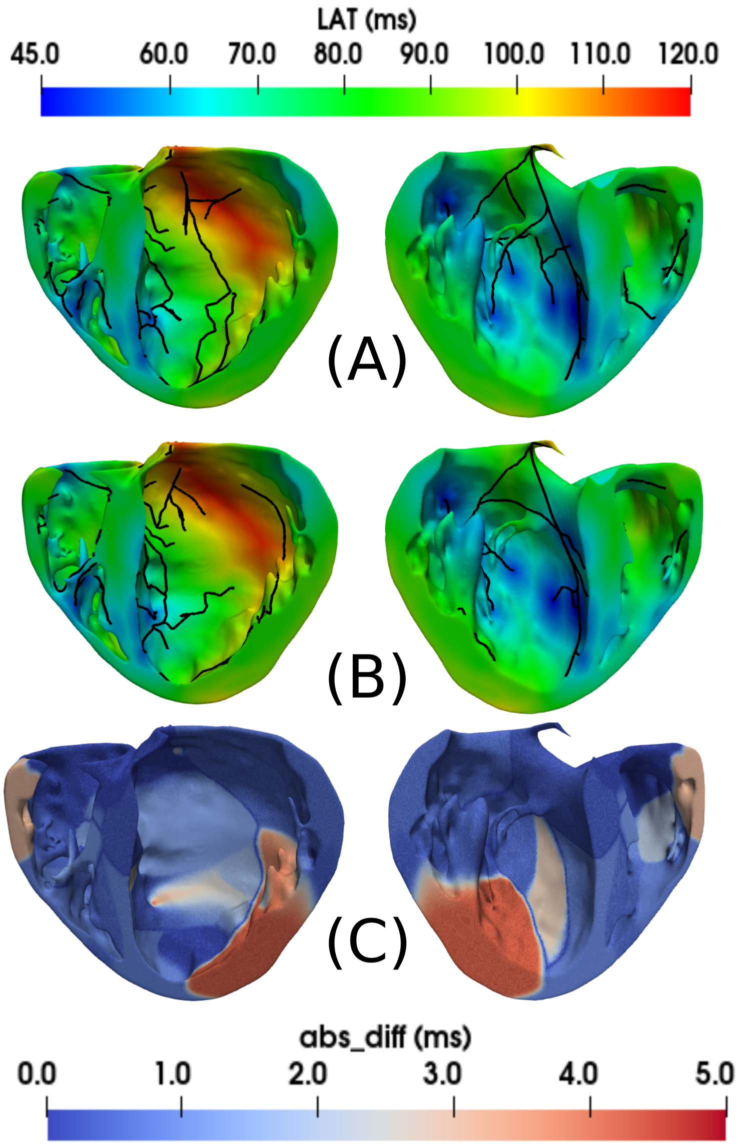

Figure S6 . Results for the LAT maps for the best (panel A), and worst (panel B), PNs generated by our method considering the patient-specific mesh alongside the absolute difference LAT map (panel C) calculated by the difference between the two LAT maps. The topology of the PNs is illustrated in black color.

A.7 Method description

The detailed main program of the method depicted in **Algorithm 1**, starts by receiving as an input: a set of points S with locations for the terminal branches and an additional set of active PVJ points, an intermediate points cost function CF_i, an active PVJ points cost function CF_a, maximum number of feasible segments to be considered in the intermediate point cost function N_i, maximum number of feasible segments to be considered in the active cost function N_a, connection rate constant L_{rate}, LAT error tolerance for an active PVJ connection L_{error}, characteristic length of the domain l_d, proximal location of the root branch

x_{prox} and an optional *initial PN* to be considered as starting root. The main parameters of the method are detailed in Table S9.

Algorithm 1: Main program.

Data: S, x_{prox}, [*initial PN*].
Input parameters: CF_i, CF_a, N_i, N_a, l_d, L_{rate}, L_{error}
Result: Purkinje network generated within the set of points S.

$S_i \leftarrow$ *PreProcessing*(S, x_{prox}); # **Algorithm 2**
$S_a \leftarrow$ *ExtractActivePVJ*(S); # **Algorithm 3**
$k_{term} \leftarrow$ *RootPlacement*(S_i, l_d, x_{prox}, [*initial PN*]); # **Algorithm 4**
while (*not pass one time through* S_i) **do**
  $x_{term} \leftarrow$ *GenerateTerminal*(S_i, N_i, CF_i, l_d, k_{term}); # **Algorithm 8**
  Include x_{term} in the tree;
  $k_{term} \leftarrow k_{term} + 1$;
  if (k_{term} % $L_{rate} == 0$) **then**
    AttemptPVJConnection(S_a, N_a, CF_a, L_{error}, l_d, k_{term}); # **Algorithm 10**
  end
end
PostProcessing(S_a, N_a, CF_a, L_{error}, l_d, k_{term}); # **Algorithm 13**
Compute metrics and save network topology to a file;

Parameter name	Description
x_{prox}	Proximal location of the root branch
S_i	Intermediate point set
S_a	Active point set (PVJs)
N_i	Maximum number of feasible segments to be considered in the intermediate point cost function
N_a	Maximum number of feasible segments to be considered in the active point cost function
CF_i	Intermediate points cost function
CF_a	Active points cost function
L_{rate}	Connection rate
L_{error}	LAT error tolerance for an active PVJ connection
l_d	Characteristic length of the domain
k_{term}	Current number of terminals in the tree
d_{thresh}	Distance criterion threshold

Table S9 . Main parameters of the method.

A.7.1 Pre-processing steps

The *PreProcessing* subroutine described in **Algorithm 2** and illustrated in Figure S7A, is responsible for remapping and filtering a percentage of the points within the input set S. The result of this procedure is the intermediate point set S_i that is utilized to generate the intermediate branches of the growing tree. The remapping of the points based on the proximal location of the root branch x_{prox}. A sphere centered at the given proximal root position and with an initial radius $r_0 = 0.01mm$ grows in intervals of $r_{inc} = 0.05mm$ until all the points in the set S are covered. At each iteration, the points within the growing sphere have their indexes renumbered consecutively. In addition, the amount of intermediate points that the method will use is filtered by 1% of the total number of intermediate points inside S. We randomly select intermediate points inside S until the prescribed quantity is achieved. Ultimately, the result of this process is a new set S_i, which is the intermediate cloud of points that the

method mainly utilizes.

Algorithm 2: *PreProcessing* subroutine.

Data: S, x_{prox}, **global** $r_0 = 0.01mm$, **global** $r_{inc} = 0.05mm$.
Result: Intermediate cloud of points S_i.

$r \leftarrow r_0$;
$S_i \leftarrow \emptyset$;
while $(|S_i| < |S|)$ **do**
    for $i \in \{0, \ldots, |S|\}$ **do**
        $x \leftarrow S[i]$;
        $dist \leftarrow ||x - x_{prox}||$;
        if $(dist < r)$ **then**
            Append x to S_i;
            Erase x from S;
        end
    end
    $r \leftarrow r + r_{inc}$;
end
$S_i \leftarrow$ Filter 1% percentage of the intermediate points inside set S_i;
return S_i;

The *ExtractActivePVJ* subroutine presented in **Algorithm 3** and illustrated in Figure S7B, manages the set S by extracting the points that are considered active PVJs. In particular, each active PVJ must have an associated LAT value as a reference for comparison. With this value, we sort the active PVJ points using the *QuickSort* algorithm[7].

Algorithm 3: *ExtractActivePVJ* subroutine.

Data: S.
Result: Active cloud of points S_a sorted by their LAT.

$S_a \leftarrow \emptyset$;
for $i \in \{0, \ldots, |S|\}$ **do**
    if $(S[i]$ *is active*$)$ **then**
        $x \leftarrow S[i]$;
        $lat \leftarrow S[i].LAT$;
        Append the pair (x, lat) to S_a;
    end
end
Apply *QuickSort* algorithm on S_a using lat as comparison parameter;
return S_a;

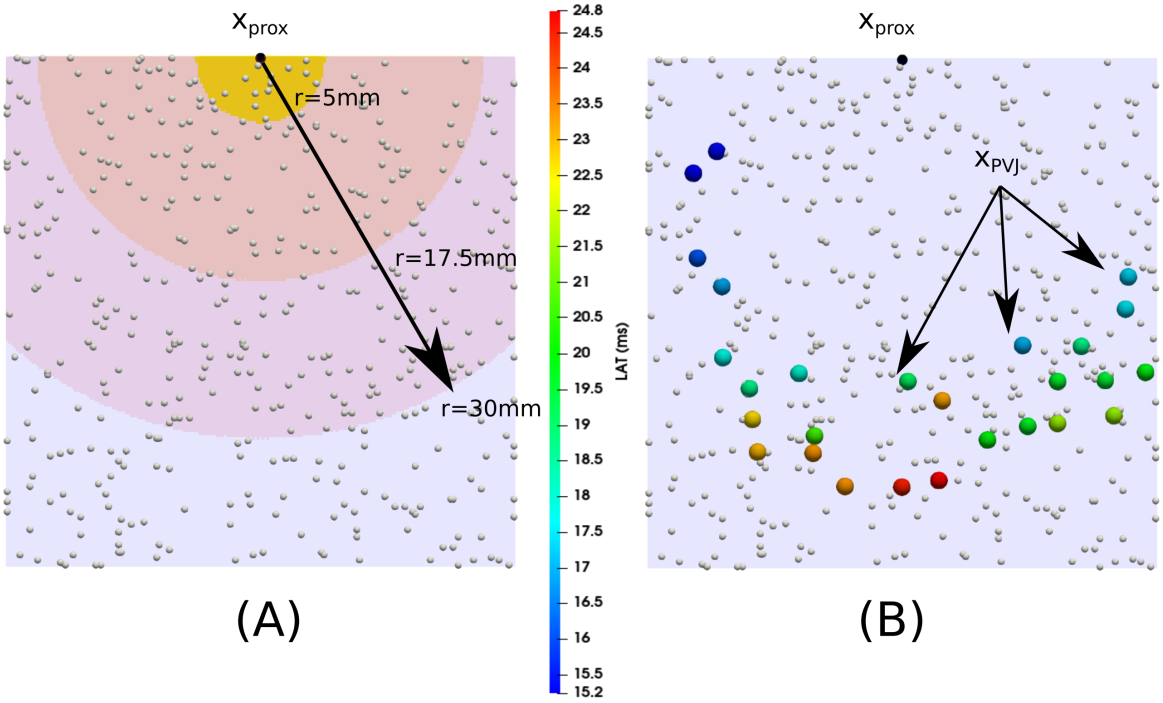

Figure S7 . Illustration of the *PreProcessing* and *ExtractActivePVJ* subroutines, where the *PreProcessing* function is shown in panel (A) and *ExtractActivePVJ* is depicted in panel (B). In panel (A), the remapped and filtered intermediate points S_i are highlighted by white spheres. In addition, the evolution of the growing sphere used to remap the points in set S is illustrated by the distinct values of its radius r. In panel (B), the intermediate points set S_i is highlighted by white spheres, while the active points set S_a is highlighted by colored spheres using the LAT colormap.

A.7.2 Root placement

The *RootPlacement* subroutine detailed in **Algorithm 4**, is responsible for generating or loading the initial root of the PN. The method starts by constructing the root branch, in which, given the proximal location x_{prox}, a distal location x_{dist} is randomly selected from the intermediate point set S_i and must attend a distance criterion. After selecting a feasible position, a geodesic pathway which connects x_{prox} to x_{dist} is constructed. Alternatively, the user can also provide an initial root structure described

in a PN topology format file that can be used to initialize the root of the method.

Algorithm 4: *RootPlacement* subroutine.

Data: S_i, l_d, x_{prox}, [*initial PN*])
Result: Initial state of the Purkinje network.

if *(initial PN is given)* **then**
  $k_{term} \leftarrow$ Load initial PN topology from file;
else
  $is\_root\_ok \leftarrow False$;
  $counter \leftarrow 0$;
  $d_{thresh} \leftarrow l_d$;
  while $(is\_root\_ok == False)$ **do**
    $x_{dist} \leftarrow$ *SortPoint*(S_i); # **Algorithm 5**
    $d \leftarrow ||x_{dist} - x_{prox}||$;
    if $(d >= d_{thresh})$ **then**
      $is\_root\_ok = True$;
    else
      $counter \leftarrow counter + 1$;
    end
    if $(counter > 8)$ **then**
      $d_{thresh} \leftarrow d_{thresh} * 0.95$;
    end
  end
  Geodesic pathway: Build a geodesic path from x_{prox} to x_{dist}
  $k_{term} \leftarrow 1$;
end
return k_{term};

The subroutine *SortPoint* described in **Algorithm 5** is responsible for selecting a point from the intermediate point set S_i. The points from the intermediate point set are selected sequentially, and to allow variability in the PNs, this selection is made using different gap values. This gap value is defined as the difference between the previous and current index selected in S_i. This value is calculated by taking the modulo between a random integer number, computed using the default *rand* function from the *C*++ standard library, and a fixed offset parameter, which was defined as 4 in this work. To avoid an index out of bounds error, we always apply a modulo operation between the next index and the total number of intermediate points in set S_i. An important aspect of this function is that when the *next_id* variable returns to the start of the S_i array, the stop criterion flag of the method's main loop is activated.

Algorithm 5: *SortPoint* subroutine.

Data: S_i, **global** *prev_id*
Result: Index of the next point to be selected in the set S_i.

$k \leftarrow rand()\%4 + 1$;
$next\_id \leftarrow (prev\_id + k)\%|S_i|$;
while ($S_i[next\_id]$ *is taken*) **do**
  $prev\_id \leftarrow next\_id$;
  $k \leftarrow rand()\%4 + 1$;
  $next\_id \leftarrow (prev\_id + k)\%|S_i|$;
end
return $S_i[next\_id]$;

A.7.3 Distance criterion

The subroutine *DistanceCriterion* depicted in **Algorithm 6**, controls if all segments in set P_s attend the distance criterion for a given x_{term} position.

Algorithm 6: *DistanceCriterion* subroutine.

Data: x_{term}, d_{thresh}, P_s
Result: Return *True* if all segments in set P_s attend the criterion or *False* otherwise.
for $i \in \{0, \ldots, |P_s|\}$ **do**
  $u \leftarrow x_p(i) - x_d(i)$;
  $v \leftarrow x_{term} - x_d(i)$;
  $w \leftarrow x_{term} - x_p(i)$;
  $d_i \leftarrow u.v/||u||^2$; # **scalar product**
  if *(* $d_i >= 0$ ***and*** $d_i <= 1$ *)* **then**
    $d_{crit} \leftarrow ||v \times w||/||u||$; # **cross product**
  else
    $d_{crit} \leftarrow \min\{||v||, ||w||\}$;
  end
  if *(* $d_{crit} < d_{thresh}$ *)* **then**
    return *False*;
  end
end
return *True*;

A.7.4 Cost function evaluation

The *EvaluateCostFunction* subroutine detailed in **Algorithm 7** evaluates the given cost function CF for every element in the feasible segment set F_s.

Algorithm 7: *EvaluateCostFunction* subroutine.

Data: x_{term}, F_s, CF
Result: A list of evaluations E using cost function CF.
$E \leftarrow \emptyset$;
for $i \in \{0, \ldots, |F_s|\}$ **do**
  Geodesic pathway: Build geodesic pathway linking x_{dist} from segment s_i to x_{term};
  $e \leftarrow$ Evaluate CF under this PN topology;
  if *(No collision is detected)* **then**
    Append e the evaluation to E;
  end
end
return E;

A.7.5 Generation of intermediate terminals

The subroutine *GenerateTerminal* depicted in **Algorithm 8** and illustrated in Figure S8, generates a new intermediate terminal branch to the PN. The generation proceeds by sorting a point x_{term} from set S_i using the sort point function presented in **Algorithm 5**. The prospective location x_{term} is accepted if x_{term} satisfies the distance criterion, which is defined by **Algorithm 6**, and if there is otherwise we select a new point from S_i. In case the above procedure fails 10 times, i.e., $d_{crit} < d_{thresh}$, the threshold distance d_{thresh} is decreased by a factor 0.95. This is repeated until the acceptance of x_{term}.

Next, when x_{term} has been accepted as the distal end of a new terminal branch, the nearest N_i feasible segments to generate a new branch are computed and stored in set F_s using subroutine *FillFeasibleSegmentsIntermediate* which is detailed in **Algorithm 9**. After this procedure, x_{term} is temporarily connected to the nearest N_i feasible segments in F_s in the model by constructing a geodesic pathway that links the distal point of the candidate segment, x_{dist}, and x_{term}. For each candidate connection, the value of the cost function given by CF_i is obtained using **Algorithm 7**. Finally, the candidate connection with the lowest value of CF_p and whose connection does not generate a collision to other segments in the tree is adopted and made

permanent. In case all feasible segments cause collisions, we sort a different x_{term} from S_i and recheck the distance criterion.

Algorithm 8: *GenerateTerminal* subroutine.

Data: S_i, N_i, CF_i, l_d, k_{term}
Result: New terminal branch added to the Purkinje network.

$d_{thresh} \leftarrow \sqrt{l_d^2/k_{term}}$;
$tosses \leftarrow 0$;
$point\_is\_ok \leftarrow False$;
$P_s \leftarrow$ Get all segments from the PN;
while $(point\_is\_ok == False)$ **do**
    $F_s \leftarrow \emptyset$;
    $x_{term} \leftarrow SortPoint(S_i)$;
    # Algorithms 6 and 9
    if (*DistanceCriterion*(x_{term}, d_{thresh}, P_s) ***and*** *FillFeasibleSegmentsIntermediate*(x_{term}, N_i, F_s, P_s)) **then**
        $E_i \leftarrow$ *EvaluateCostFunction*(x_{term}, F_s, CF_i); **# Algorithm 7**
        if ($|E_i| > 0$) **then**
            $point\_is\_ok = True$;
        end
    else
        $tosses \leftarrow tosses + 1$;
        if ($tosses > 10$) **then**
            $tosses \leftarrow 0$;
            $d_{thresh} \leftarrow d_{thresh} * 0.95$;
        end
    end
end
Geodesic pathway: Build a branch linking x_{dist} from the segment with the best CF_i evaluation in E_i to x_{term};
return x_{term};

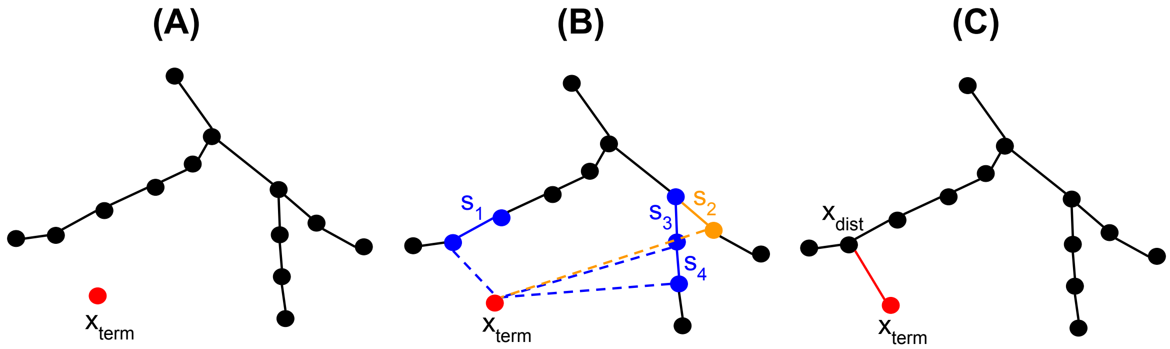

Feasible segment	Cost function evaluation
**s1**	**2**
s2	Collision!
s3	4
s4	5

→ Best evaluation

Figure S8 . Illustration of the *GenerateTerminal* subroutine described in **Algorithm 8**. In panel (A), a prospective location for a new terminal branch, x_{term}, is selected after attending the distance criterion. Next, in panel (B), the N_i feasible segments in F_s are evaluated by the cost function CF_i and ranked in a table. After the evaluation step, segment 2 is unfeasible for the connection since it generates a collision in the PN. Segment 1 returns the minimum value for the cost function and is considered the best evaluation. Finally, in panel (C), a new terminal branch is constructed by linking the distal position of segment 1, x_{dist}, to the location x_{term}.

The *FillFeasibleSegmentsIntermediate* subroutine as presented in **Algorithm 9** is responsible for populating the F_s set for the intermediate point cost function with the nearest N_i segments to the x_{term} position. This is done by passing through all the segments in the current PN and computing the euclidean distance between the middle position of each segment, x_M, and x_{term}.

Algorithm 9: *FillFeasibleSegmentsIntermediate* subroutine.

Data: x_{term}, N_i, F_s, P_s
Result: Fill the feasible segments set F_s to connect x_{term}.
for $i \in \{0,\dots,|P_s|\}$ **do**
  $dist \leftarrow ||x_{term} - x_M(s_i)||$;
  Append $(i, dist)$ to F_s;
end
Apply *QuickSort* algorithm on F_s using *dist* as comparison parameter;
return $F_s[0:N_i]$;

A.7.6 Generation of active terminals

The *AttemptPVJConnection* subroutine described in **Algorithm 10**, attempt to connect all the remaining active PVJ points from set S_a to the current PN. This procedure is repeated until no new active PVJ can be connected to the structure.

Algorithm 10: *AttemptPVJConnection* subroutine.

Data: S_a, N_a, CF_a, L_{error}, l_d, k_{term}
Result: Try to connect remaining PVJs inside S_a with new terminal branches.
repeat
  for $i \in \{0,\dots,|S_a|\}$ **do**
    $x_{PVJ} \leftarrow S_a(i)$;
    if *(x_{PVJ} is not connected)* **then**
      AttemptGeneratePVJ(x_{PVJ}, N_a, CF_a, L_{error}, l_d, k_{term}); # **Algorithm 11**
    end
  end
until *(No new PVJ is connected)*

The *AttemptGeneratePVJ* subroutine described in **Algorithm 11** and illustrated by Figure S9, respectively, attempt to connect an active PVJ, x_{PVJ}, by generating a new terminal branch. The branch is only made permanent if the absolute LAT error between the best candidate branch and the reference LAT value of the target active PVJ is less or equal to L_{error}. Otherwise, the PVJ returns to set S_a and the entire branch is pruned.

Algorithm 11: *AttemptGeneratePVJ* subroutine.

Data: x_{PVJ}, N_a, CF_a, L_{error}, l_d, k_{term}
Result: Try to connect the x_{PVJ} within the LAT error tolerance with a new branch.
$d_{thresh} \leftarrow \sqrt{l_d^2/k_{term}}$;
$F_s \leftarrow \emptyset$;
$P_s \leftarrow$ Get all segments from the PN;
# **Algorithms 6 and 12**
if *(DistanceCriterion(x_{PVJ}, d_{thresh}, P_s)* ***and*** *FillFeasibleSegmentsActive(x_{PVJ}, N_a, F_s, P_s))* **then**
  $E_a \leftarrow$ *EvaluateCostFunction(x_{PVJ}, F_s, CF_a)*; # **Algorithm 7**
  if *($|E_a| > 0$)* **then**
    Geodesic pathway: Build a branch linking x_{dist} from the segment with the best CF_a evaluation in E_a to x_{PVJ};
    if *(x_{PVJ} is not connected with an error less than L_{error})* **then**
      Prune the previous branch with x_{PVJ};
    else
      Tag x_{PVJ} as connected;
    end
  end
end

The *FillFeasibleSegmentsActive* subroutine as presented in **Algorithm 12** is responsible for populating the F_s set for the active cost function with the nearest N_a segments to the x_{PVJ} position. This is done by passing through all the segments in the current PN and computing an approximation of the LAT error to the x_{PVJ} reference value. This approximation is calculated by

the sum of the current LAT of a given segment s_i and the LAT given by the line that links the middle position x_M of segment s_i to x_{PVJ} using the cable equation.

Algorithm 12: *FillFeasibleSegmentsActive* subroutine.

Data: x_{PVJ}, N_a, F_s, P_s
Result: Fill the feasible segments set F_s to connect x_{term}.
for $i \in \{0, \ldots, |P_s|\}$ **do**
  $dist \leftarrow ||x_{PVJ} - x_M(s_i)||$;
  $lat \leftarrow s_i.LAT + (dist/s_i.cv)$;
  $error \leftarrow |x_{PVJ}.LAT - lat|$;
  Append $(i, error)$ to F_s;
end
Apply *QuickSort* algorithm on F_s using *error* as comparison parameter;
return $F_s[0 : N_a]$;

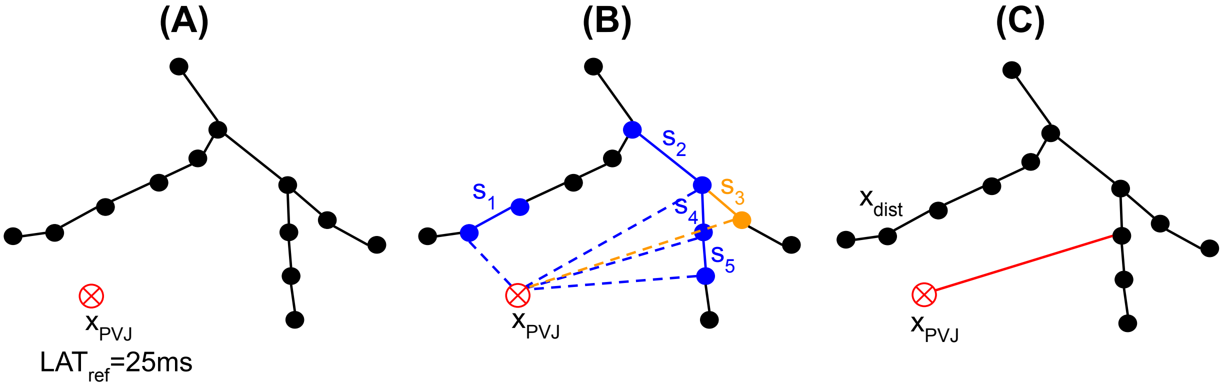

Feasible segment	Cost function evaluation
s1	|25-30|=5ms
s2	|25-21|=4ms
s3	Collision!
**s4**	**|25-24|=1ms**
s5	|25-27|=2ms

→ Best evaluation (s4)

Figure S9 . Illustration of the *AttemptGeneratePVJ* subroutine described in **Algorithm 11**. In panel (A), a prospective location for a new PVJ branch, x_{PVJ}, is selected after attending the distance criterion. Next, in panel (B), N_a feasible segments are evaluated by the cost function CF_a and ranked in a table. After the evaluation step, segment 3 is unfeasible for the connection since it generates a collision in the PN and segment 4 returns the minimum value for the cost function and is within the LAT error tolerance $L_{error} = 2ms$, for that reason is considered the best evaluation. Finally, in panel (C), a new terminal PVJ branch is constructed by linking the distal position of segment 4, x_{dist}, to the location x_{PVJ}.

A.7.7 Post-processing steps

In the case there are remaining active PVJs to be connected after the main loop, we apply a post-processing step described and illustrated in **Algorithm 13** and Figure S10, respectively. The first step in this procedure is to prune all intermediate branches of the tree. A branch is considered to be intermediate if it is not directly part of a pathway that links an active PVJ to the root. Next, the LAT error tolerance constraint is dropped by setting $L_{error} = \infty$, and we attempt to connect all unconnected PVJs in S_a using the pruned tree. The first N_a feasible segments sorted by the LAT error are evaluated using cost function CF_a and the segment with the best evaluation is connected using a geodesic pathway to x_{PVJ}.

However, after this procedure some PVJs still could not be connected due to the distance criterion. This scenario could happen if x_{PVJ} is already too close to the current tree. In this particular case, we drop the distance criterion for these PVJs and attempt to connect the point with a feasible segment which returns the minimum LAT error using a geodesic pathway. If there are no geodesic pathway possible for x_{PVJ}, we consider the 5 closest segments by distance to x_{PVJ} and force its connection to

the one that returns the minimum LAT error using a straight line, regardless of any restriction. After this step, all the active PVJs that the user-specified are connected and the geometric and activation metrics are computed in this topology, which is referred to as *minimum network*.

Algorithm 13: *PostProcessing* subroutine.

Data: $S_a, N_a, CF_a, L_{error}, l_d, k_{term}$
Result: Minimum Purkinje network with all active PVJs connected.

Prune intermediate branches;
Remove the LAT error tolerance constraint for an active PVJ connection;
for $i \in \{0, \ldots, |S_a|\}$ **do**
  $x_{PVJ} \leftarrow S_a[i]$;
  if *(*x_{PVJ} *is not connected)* **then**
    AttemptGeneratePVJ($x_{PVJ}, N_a, CF_a, \infty, l_d, k_{term}$*)*; # **Algorithm 11**
    if *(*x_{PVJ} *is not connected)* **then**
      Remove the distance criterion for x_{PVJ};
      $F_s \leftarrow \emptyset$;
      $P_s \leftarrow$ Get all segments from the PN;
      FillFeasibleSegmentsActive(x_{PVJ}, N_a, F_s, P_s*)* # **Algorithm 12**
      $E_a \leftarrow$ *EvaluateCostFunction(*x_{PVJ}, F_s, CF_a*)*; # **Algorithm 7**
      if *(* $|E_a| > 0$ *)* **then**
        Geodesic pathway: Build a branch linking x_{dist} from the segment with the best CF_a evaluation in E_a to x_{PVJ};
      else
        $F_s \leftarrow$ Get the 5 closest segment by distance to x_{PVJ};
        Straight line: Build a branch linking x_{dist} from the segment with the best CF_a evaluation in F_s to x_{PVJ};
      end
    end
  end
end

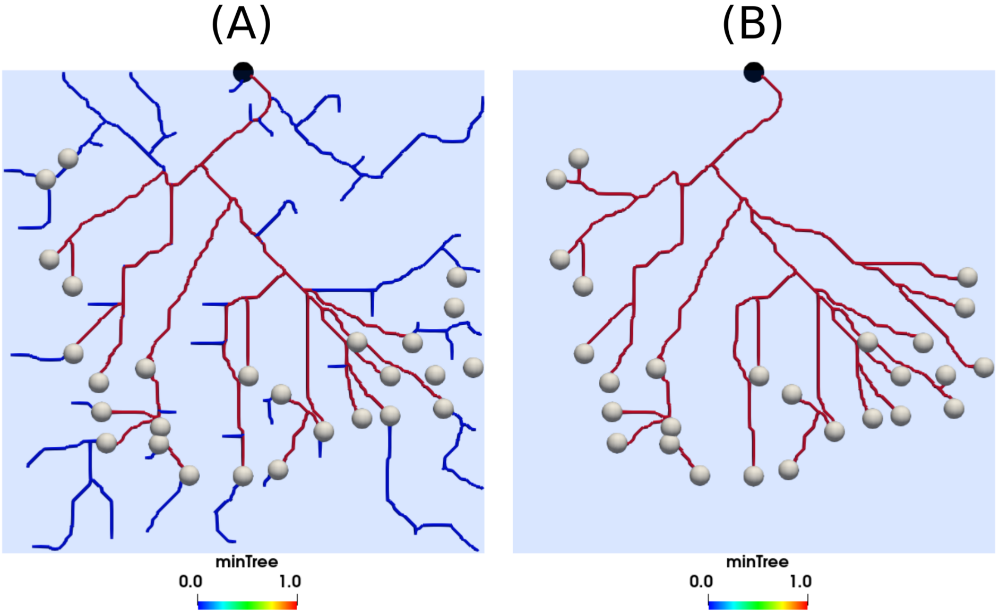

Figure S10 . Illustration of the *PostProcessing* subroutine described in **Algorithm 13**, where the difference between intermediate and active branches is shown in panel (A) and the resultant *minimum network* is depicted in panel (B). In panel (A), the intermediate branches are colored in blue, while the active branches are in red. The black dot highlights the root node, and the active PVJs are referenced as white spheres.

A.8 Finite Volume Method for solving the monodomain model in Purkinje-coupled simulations

The monodomain model is represented by a reaction-diffusion equation given by:

$$\beta\left(C_m\frac{\partial V}{\partial t}+I_{ion}(V,\eta)\right)=\nabla.(\sigma\nabla V), \tag{8}$$

The Finite Volume Method (FVM) can be used to solve the monodomain model. A common technique that solvers of cardiac electrophysiology perform when solving the monodomain model is to use an operator *splitting* to divide the equation in two distinct problems: a parabolic PDE associated to the diffusion part and a non-linear system of ODEs related to the reaction part of the model[8–10].

In this work we solve the monodomain model for two different domains: the Purkinje and Ventricular. Within this context, whenever we are considering the transmembrane potential for the Purkinje domain we denote, V_{PK}, and for the Ventricular domain, V_{VT}. In summary, to advance the simulation by one timestep we need to solve four problems, two linear system associated to the diffusion part of the Purkinje and Ventricular domains, and two non-linear system of ODEs related to the reaction part of the Purkinje and Ventricular cellular models.

Additionally, it is important to remember that the Purkinje and Ventricular domains are coupled at the PVJ sites. For that reason we need to consider two extra currents: I_{PVJ}^{purk} and I_{PVJ}^{tiss}. These two currents describe the fluxes coming from the Ventricular to the Purkinje domains, and from the Purkinje to the Ventricular ones, respectively, are given by equations (6) and (7). In the following equations we describe the reaction and diffusion parts of the monodomain model for both the Purkinje and Ventricular domains after applying a time discretization using an implicit Euler scheme.

Firstly, we solve the reaction part of the Purkinje cellular model associated to equations (9) and (10), then we update the right-hand side of the Purkinje linear system which is related to the diffusion part of the model given by (11). Then, we compute the PVJ coupling current from the tissue to the Purkinje by adding the I_{PVJ}^{purk} current coming from the coupled ventricular cells to the terminal Purkinje cells using equation (7), updating their transmembrane potential. During this step the current I_{PVJ}^{purk} is added together with the fluxes from the faces of a control volume when the term $\nabla.(\sigma\nabla V_{PK}^{n+1/2})$ in equation (11) is discretized using the FVM. Next, we solve the Purkinje diffusion linear system given by (11).

$$C_m\left(\frac{V_{PK}^{n+1/4}-V_{PK}^{n}}{\Delta t}\right)=-I_{ion_{PK}}(V_{PK}^{n},\eta_{PK}^{n}), \tag{9}$$

$$\frac{\partial\eta_{PK}}{\partial t}=f_{PK}(V_{PK}^{n},\eta_{PK}^{n}), \tag{10}$$

$$C_m\left(\frac{V_{PK}^{n+1/2}-V_{PK}^{n+1/4}}{\Delta t}\right)=\frac{\nabla.(\sigma_{PK}\nabla V_{PK}^{n+1/2})+\lambda I_{PVJ}^{purk}}{\beta}, \tag{11}$$

where V_{PK} is the transmembrane potential for the Purkinje domain, $I_{ion_{PK}}$ is the total ionic current for the Purkinje cellular model that may also depend on Purkinje gating variables η_{PK}, I_{PVJ}^{purk} is the PVJ coupling current coming from the coupled ventricular cells to the terminal Purkinje cells, λ is a binary variable that is equal to zero when a ventricular cell is not coupled to a Purkinje cell and one otherwise, σ_{PK} is the monodomain Purkinje conductivity, β is the surface-volume ratio and C_m is the membrane capacitance.

Secondly, we solve the Ventricular reaction part given by equations (12) and (13). Next, we update the right-hand side of the Ventricular linear system. Next, we compute the PVJ coupling current from the Purkinje to the tissue by adding the I_{PVJ}^{tiss} current coming from the terminal Purkinje cells to the coupled ventricular cells using equation (6), updating their transmembrane potentials. Similarly, the current I_{PVJ}^{purk} is added together with the fluxes from the faces of a control volume when the term $\nabla.(\sigma\nabla V_{VT}^{n+1})$ in equation (14) is discretized using the FVM. Finally, we solve the Ventricular diffusion linear system associated to equation (14) advancing the simulation by one timestep.

$$C_m\left(\frac{V_{VT}^{n+3/4}-V_{VT}^{n+1/2}}{\Delta t}\right)=-I_{ion_{VT}}(V_{VT}^{n+1/2},\eta_{VT}^{n+1/2}), \tag{12}$$

$$\frac{\partial\eta_{VT}}{\partial t}=f_{VT}(V_{VT}^{n+1/2},\eta_{VT}^{n+1/2}), \tag{13}$$

$$C_m\left(\frac{V_{VT}^{n+1}-V_{VT}^{n+3/4}}{\Delta t}\right)=\frac{\nabla.(\sigma_{VT}\nabla V_{VT}^{n+1})+\alpha I_{PVJ}^{tiss}}{\beta}, \tag{14}$$

where V_{VT} is the transmembrane potential for the Ventricular domain, $I_{ion_{VT}}$ is the total ionic current for the Ventricular cellular model that may also depend on Ventricular gating variables η_{VT}, I_{PVJ}^{tiss} is the PVJ coupling current coming from the terminal Purkinje cells to the coupled ventricular cells, α is a binary variable that is equal to zero when a Purkinje cell is not coupled to a ventricular cell and one otherwise and σ_{VT} is the monodomain Ventricular conductivity tensor.

References

1. Costabal, F. S., Hurtado, D. E. & Kuhl, E. Generating Purkinje networks in the human heart. *J. Biomech.* **49**, 2455–2465 (2016).
2. Trovato, C. *et al.* Human Purkinje in silico model enables mechanistic investigations into automaticity and pro-arrhythmic abnormalities. *J. Mol. Cell. Cardiol.* **142**, 24–38 (2020).
3. Tomek, J., Bueno-Orovio, A. & Rodriguez, B. ToR-ORd-dynCl: an update of the ToR-ORd model of human ventricular cardiomyocyte with dynamic intracellular chloride. *BioRxiv* (2020).
4. Wiedmann, R. T., Tan, R. C. & Joyner, R. W. Discontinuous conduction at Purkinje-ventricular muscle junction. *Am. J. Physiol. Circ. Physiol.* **271**, H1507–H1516 (1996).
5. Liu, B. R. & Cherry, E. M. Image-based structural modeling of the cardiac Purkinje network. *BioMed Res. Int.* (2015).
6. Ulysses, J. N. *et al.* An optimization-based algorithm for the construction of cardiac Purkinje network models. *IEEE Transactions on Biomed. Eng.* **65**, 2760–2768 (2018).
7. Cormen, T. H., Leiserson, C. E., Rivest, R. L. & Stein, C. *Introduction to algorithms* (MIT press, 2022).
8. Qu, Z. & Garfinkel, A. An advanced algorithm for solving partial differential equation in cardiac conduction. *IEEE Transactions on Biomed. Eng.* **46**, 1166–1168 (1999).
9. Sachetto Oliveira, R. *et al.* Performance evaluation of GPU parallelization, space-time adaptive algorithms, and their combination for simulating cardiac electrophysiology. *Int. J. for Numer. Methods Biomed. Eng.* **34**, e2913 (2018).
10. Sundnes, J. *et al.* On the computational complexity of the bidomain and the monodomain models of electrophysiology. *Annals biomedical engineering* **34**, 1088–1097 (2006).
